# Blood-feeding adaptations and virome assessment of the poultry red mite *Dermanyssus gallinae* guided by RNA-seq

**DOI:** 10.1038/s42003-023-04907-x

**Published:** 2023-05-13

**Authors:** José M. Ribeiro, David Hartmann, Pavla Bartošová-Sojková, Humberto Debat, Martin Moos, Petr Šimek, Jiří Fara, Martin Palus, Matěj Kučera, Ondřej Hajdušek, Daniel Sojka, Petr Kopáček, Jan Perner

**Affiliations:** 1 Laboratory of Malaria and Vector Research, National Institute of Allergy and Infectious Diseases, Bethesda, MD USA; 2 Institute of Parasitology, Biology Centre, Czech Academy of Sciences, 37005 České Budějovice, Czech Republic; 3 Instituto de Patología Vegetal, Centro de Investigaciones Agropecuarias, Instituto Nacional de Tecnología Agropecuaria (IPAVE-CIAP-INTA), Córdoba, Argentina; 4 Institute of Entomology, Biology Centre, Czech Academy of Sciences, 37005 České Budějovice, Czech Republic; 5 International Poultry Testing Station Ústrašice, Ústrašice, Czech Republic

**Keywords:** Parasitology, Evolutionary developmental biology, Data mining

## Abstract

*Dermanyssus gallinae* is a blood-feeding mite that parasitises wild birds and farmed poultry. Its remarkably swift processing of blood, together with the capacity to blood-feed during most developmental stages, makes this mite a highly debilitating pest. To identify specific adaptations to digestion of a haemoglobin-rich diet, we constructed and compared transcriptomes from starved and blood-fed stages of the parasite and identified midgut-enriched transcripts. We noted that midgut transcripts encoding cysteine proteases were upregulated with a blood meal. Mapping the full proteolytic apparatus, we noted a reduction in the suite of cysteine proteases, missing homologues for Cathepsin B and C. We have further identified and phylogenetically analysed three distinct transcripts encoding vitellogenins that facilitate the reproductive capacity of the mites. We also fully mapped transcripts for haem biosynthesis and the ferritin-based system of iron storage and inter-tissue trafficking. Additionally, we identified transcripts encoding proteins implicated in immune signalling (Toll and IMD pathways) and activity (defensins and thioester-containing proteins), RNAi, and ion channelling (with targets for commercial acaricides such as Fluralaner, Fipronil, and Ivermectin). Viral sequences were filtered from the Illumina reads and we described, in part, the RNA-virome of *D. gallinae* with identification of a novel virus, Red mite quaranjavirus 1.

## Abstract (web summary)

Genes associated with the consumption of blood and iron storage are identified in the poultry red mite, along with presentation of a partial virome.

## Introduction

The *Dermanyssus* mites are blood-feeding ectoparasites of birds^1^. The poultry red mite (*D. gallinae*) is a global pest in layer houses for both domestic and commercial-intensive egg production^2–4^, part of an important and ever-increasing global market^5^. *D. gallinae* mites have a very short life cycle, going from juvenile to mature adult stages within one week. The necessity to blood feed for most developmental stages and the swift reproductive dynamics makes *D. gallinae* a highly irritating and troublesome pest. During blood-feeding, *D. gallinae* mites can transmit several significant animal pathogens to their hosts^6^, including some that are zoonotic^7^. Although a large number of viruses and bacteria have been found associated with *D. gallinae*, its capacity to act as a vector or reservoir has been supported experimentally for only a few pathogens^8–10^. This is especially alarming for transmission of *Salmonella* spp.^10^, causing egg-associated salmonellosis and fowl typhoid disease^11^, as well as the spread of avian influenza A virus^12^. *D. gallinae* has also been associated with several other bacterial and viral species, with proof awaiting experimental confirmation of its vectorial capacity^2,10,13,14^.

Despite general knowledge of the global impact of mite infestation on hen welfare in egg-laying production, our understanding of molecular processes enabling swift and repetitive blood-feeding, blood digestion, development, and reproduction remains scarce. Previous transcriptomic studies described transcriptomes of whole bodies of *D. gallinae* mites, mostly in a developmental stage- or feeding-status-specific manner^15–18^. To further increase our knowledge, we aimed to identify midgut-specific transcripts encoding proteins that are key for successful blood feeding and digestion. To achieve that, we compared newly sequenced and assembled transcriptomes, with subsequent selection for transcripts enriched in blood-fed over unfed mites, with clear gut-specific expression. Special attention was paid to processes inherent for successful blood-feeders, including enzymatic digestion of host blood proteins, haem and iron biology, vitellogenesis, and innate immunity. The RNA-seq data were then verified by RT-qPCR on cDNA sets prepared from multiple independent biological replicates. In addition, we complemented the RNA-seq data analysis with several bioassays, whereby small-molecule inhibitors were introduced to the mite by ex vivo membrane feeding or microinjection into its haemocoel in order to understand the importance of selected molecules and pathways. Additionally, Illumina reads of viral origin was filtered out and used for a partial assembly of the mite RNA virome.

## Results

### *D. gallinae* transcriptome assembly and data availability

**Fig. 1:**
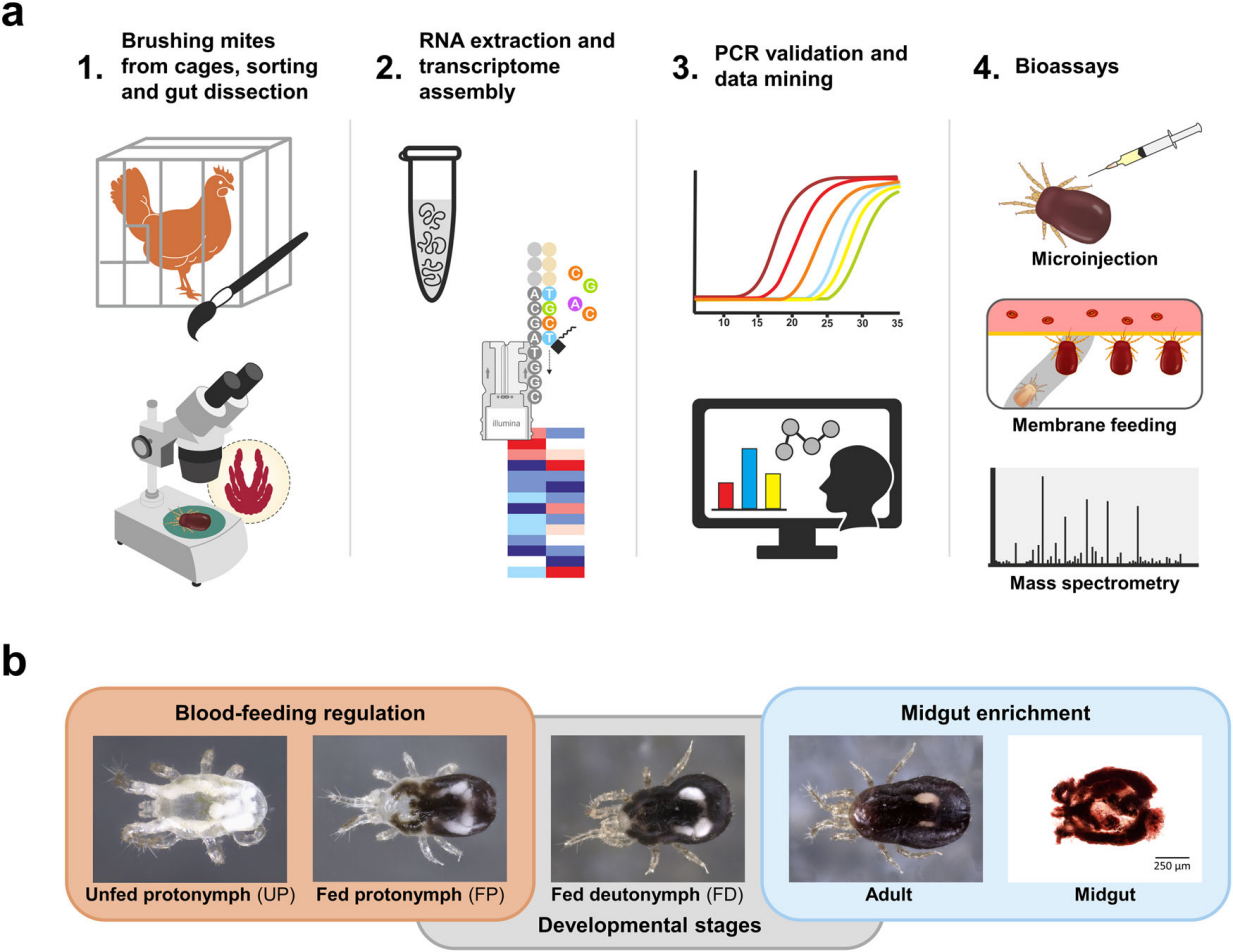
Experimental workflow and illustration of used *D. gallinae* mite’s developmental stages. **a** Schematic illustration of the experimental workflow undertaken in this work. **b** Photomicrographic illustration of representative individuals sorted by developmental stage.

Transcriptome composition of four developmental stages of thirty individuals of *D. gallinae* mites or their micro-dissected midguts were investigated in this work (Fig. 1a) by de novo assembly of Illumina RNA-seq reads. Specifically, we constructed five RNA-seq libraries derived from whole bodies of unfed protonymphs (UP, starved individuals) and blood-fed protonymphs (FP), i.e. the only developmental stage that has both naive unfed and blood-fed individuals, allowing the assessment of transcript regulation in response to host blood feeding (Fig. 1b). These were complemented by fed deutonymphs and adults, and midguts dissected from blood-fed adults (Fig. 1b). For each library, >54 M reads with Phred Scores of Q30 ≥ 93.90% were obtained, and these were assembled into 85,117 contigs (Supplementary Table S1). Following their annotation, bacterial contaminants were removed (Supplementary Table S2). As *D. gallinae* mites were fed chicken blood, consisting of nucleated white and red cells, 4% of total reads were of chicken origin, out of which (79%) were found, as expected, in the midgut transcriptome (Supplementary Fig. S1). The identified chicken sequences were filtered out and excluded from the following analyses. A total of 18,101 *D. gallinae*-specific contigs were deposited at the NCBI server as BioProject PRJNA597301 and Transcriptome Shotgun Assembly (TSA) GIFZ00000000 and are accessible through NCBI BLAST of the TSA database. The contigs are also listed in a hyper-linked Excel sheet (see “Data availability”) with available encoded predicted protein characteristics, annotations of assembled contigs according to a particular database, differential expression statistics, and predicted cellular processes. To assess the completeness of the transcriptome, we ran a BUSCO analysis of the proteome, the result of which exhibited a yield of 91.8% complete BUSCOs. The heat map generated from *D. gallinae*-specific contigs clearly indicated differences between transcriptomes of immature and adult stages and also highlighted differences in the abundance of transcripts in blood-fed over unfed mites (Supplementary Fig. S1).

### Blood-feeding stimulates the expression of midgut-specific proteases and extracellular matrix proteins

**Fig. 2:**
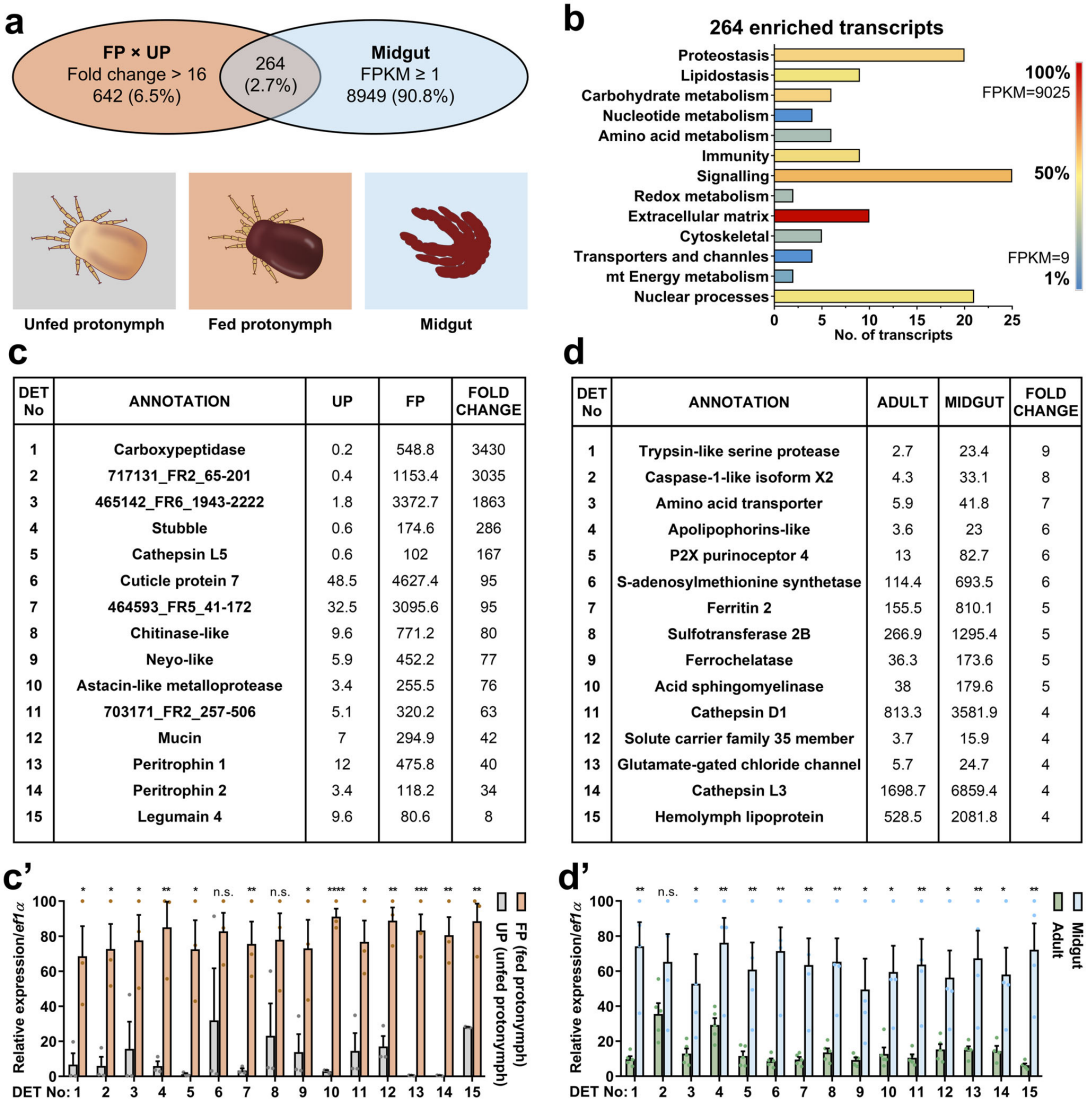
Comparative transcriptomics, with RT-qPCR validation, to identify blood digestion-associated transcripts. **a** Venn diagrams show an overlap of composition with transcripts enriched in fed protonymphs >16× FPKM values over unfed protonymphs and with transcripts of FPKM ≥ 1 in midguts. **b** The bar graph shows protein classes encoded by individual transcripts enriched by blood-feeding. Transcripts were sorted according to encoded protein class. The number of transcripts from each subclass and their FPKM values are shown. **c** The table shows top differentially expressed transcripts (DET) enriched, >16× FPKM values, in transcriptomes of blood-fed protonymphs (FP) over transcriptomes of unfed protonymphs (UP). Individual accession IDs are available in Supplementary Table S3. **c’** RT-qPCR validation of DETs identified by RNA-seq data shown in panel (**c**). Data were obtained from cDNA sets synthesised from three independent RNA isolates of unfed and blood-fed mites (*n* = 3) and normalised to *elongation factor 1* (*ef1α*). Means and SEMs are shown. **d** The table shows DETs enriched in transcriptomes of midguts over transcriptomes of whole bodies, with filters applied: Eval < e^−60^, coverage ≥ 90%, FPKM_Adults_ ≥ 2. Individual accession IDs are available in Supplementary Table S4. **d’** RT-qPCR validation of DETs identified by RNA-seq data shown in panel (**d**). Data were obtained from cDNA sets synthesised from at least four independent RNA isolates of adult females and micro-dissected midguts of adult females (*n*  ≥  4) and normalised to *ef1α*. Means and SEMs are shown. *t*-Test analyses: *p = 0.05–0.01; **p = 0.01–0.001; ***p = 0.0008; ****p < 0.0001; n.s. not significant. For the source data behind the graphs, see Supplementary Data S1.

To identify blood-feeding-associated transcripts, we have compared transcriptomes of blood-fed and UP (Fig. 2a), which developed from non-feeding larvae, i.e. a developmental stage that has not yet come into contact with blood. To identify transcripts that encode proteins key for the blood-feeding of mites, we filtered transcripts that were at least 16 times more abundant in blood-FP than in UP. These totalled 906 transcripts (Fig. 2a). These transcripts were further enriched for those having at least an FPKM value of 1 in the midgut transcriptomic library. In this way, we identified 264 transcripts (Fig. 2a), which we believe encode blood-feeding-associated proteins. Gene ontology indicates enrichment in signalling, nuclear regulation of expression, and proteostasis, with the extracellular matrix and signalling comprising the most abundant transcripts (Fig. 2b). Among the most differentially expressed transcripts (DETs) are secreted metallocarboxypeptidase (M14 protease), cysteine proteases (Cathepsin L5 and Legumain 4), serine protease (Stubble), and extracellular structural glycoproteins, such as cuticle protein, mucin, and peritrophins (Fig. 2c). To render transcript expression FPKM values amenable for statistically meaningful quantitative comparison, the transcriptomes were validated by real-time quantitative PCR (RT-qPCR) from independent templates of at least four biological replicates (see below). RT-qPCR analysis confirmed blood-feeding regulation, with most of the transcripts being significantly upregulated in blood-fed compared to unfed mites (Fig. 2c**’**). To gain further insight into midgut-specific processes, we selected differentially expressed midgut transcripts displaying a fold change in midgut expression FPKM values over adult whole bodies. Transcripts with the most profound midgut-specific expression are those encoding proteases, ferritin 2 (iron-binding protein), ferrochelatase (a terminal enzyme of haem biosynthesis), and haemolymph transporting lipoglycoprotein (Fig. 2d). These DETs were again confirmed by independent RT-qPCR (Fig. 2d**’**). These data underscore proteolytical digestion and metal homeostasis (metallostasis) as key to the success of blood-feeding of *D. gallinae* mites.

### The digestive proteolytic system of *D. gallinae* is based on Cathepsin L and Cathepsin D

**Fig. 3:**
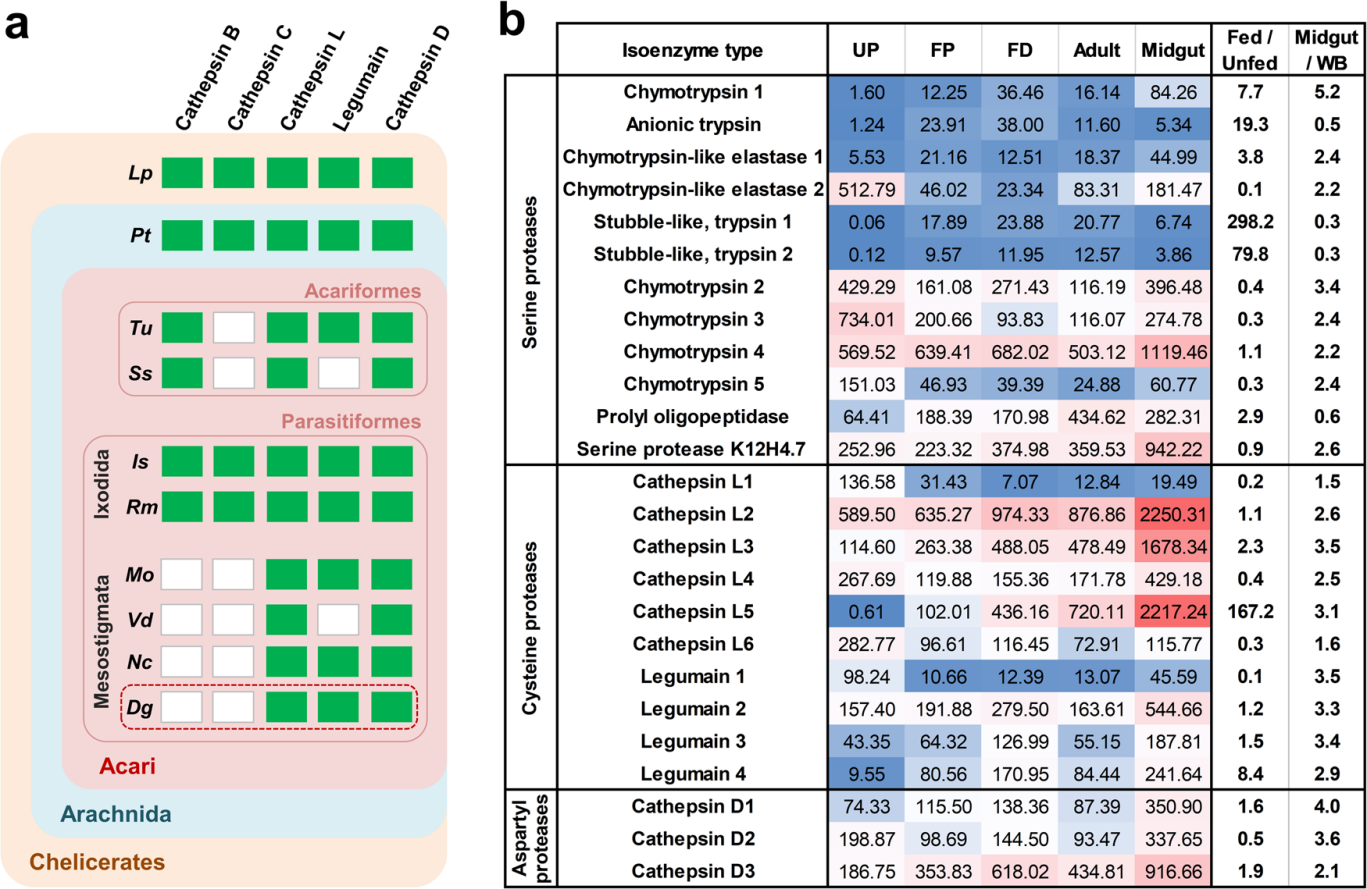
Proteases-encoding transcripts within the *D. gallinae* transcriptomes. **a** Identification of digestive protease homologues in *Dermanyssus gallinae* (*Dg*) transcriptome (highlighted by a red dashed rectangle) and representatives of Chelicerates; *Lp*, *Limulus polyphemus*; *Pt*, *Parasteatoda tepidariorum* (a representative of Araneae); *Tu*, *Tetranychus urticae*; *Ss*, *Sarcoptes scabiei*; *Is*, *Ixodes scapularis*; *Rm*, *Rhipicephalus microplus*; *Mo*, *Metaseiulus occidentalis*; *Vd*, *Varroa destructor*; *Nc*, *Neoseiulus cucumeris*. Green rectangles indicate the presence, and white rectangles indicate the absence of homologue identification with threshold *E*-values set to ≥1e^−30^. Details of Blast search set-ups and outcomes are shown in Supplementary Fig. S2. **b** A list of selected proteases and expression values (FPKM) of their mRNA transcripts across libraries derived from individual developmental stages of *D. gallinae*. Individual accession IDs are available in Supplementary Table S5. UP unfed protonymphs, FP fed protonymphs, FD fed deutonymphs, WB whole bodies. The expression variance for each transcript is indicated by colours ranging from low (blue) to high (red).

Midgut proteases clearly represent a major enzymatic suite that enables swift blood processing. To disclose a full repertoire of the *D. gallinae* digestive proteolytical system, we mined the transcriptomic libraries and identified transcripts encoding proteases of individual protease clans and families. Cysteine proteases (mainly the papain family) were found to be encoded by the majority of midgut reads identified, accounting for ~62% of all protease-encoding reads (Supplementary Fig. S2). Among the 10 cysteine proteases identified, six encoded cathepsin-l-like molecules (clan CA, C1 family) and four encoded legumains (asparaginyl endopeptidases; clan CD, C13 family) (Fig. 3a, b). Cathepsin L5 and Legumain 4 were strongly upregulated by blood-feeding and appeared to be primarily expressed in the midgut of adult *D. gallinae*, indicating their direct involvement in host blood digestion in the mite midgut. Three aspartyl proteases (AP) of cathepsin-D type (clan AA, A1 family A1) were identified, with a midgut-enriched expression pattern **(**Fig. 3b**)**. Interestingly, no homologues of key papain-like proteases cathepsins B and C (dipeptidyl peptidase I), which participate in blood digestion in ticks and other blood-feeding parasites^19,20^, were identified in the *D. gallinae* transcriptomes (Fig. 3a). Their absence seems to be a common trait among mesostigmate mites (Fig. 3a), indicating an emergence of blood feeding in *D. gallinae* mites on the molecular background of an enzymatic-repertoire devoid of Cathepsin B and C, i.e. enzymes initially thought to be indispensable for blood digestion in parasitic species^21^.

### *D. gallinae* operates haem biosynthesis and encodes both cytosolic and secreted ferritin

**Fig. 4:**
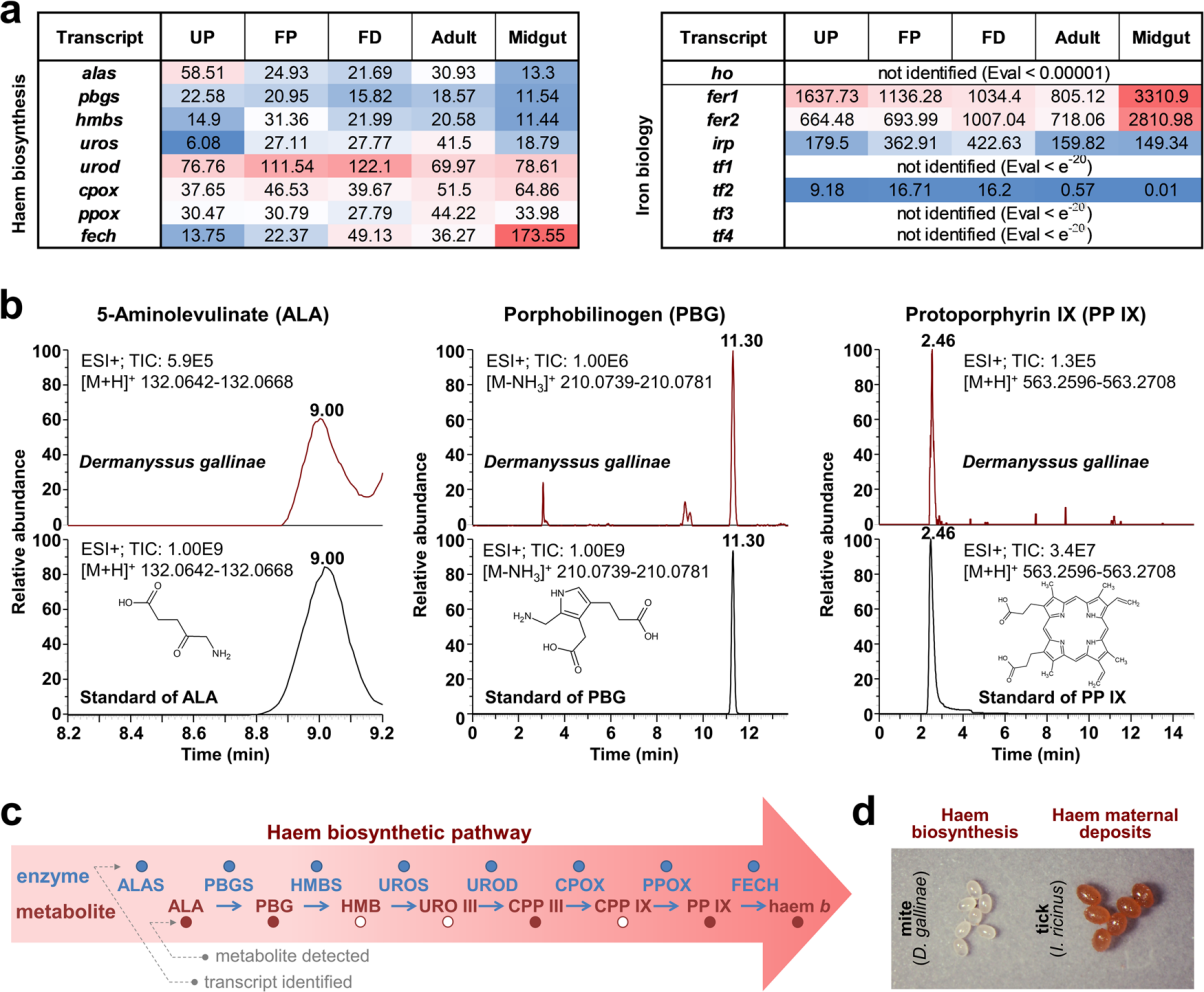
Determination of full haem biosynthesis and identification of two distinct ferritin transcripts. **a** Table of proteins participating in haem (left) or iron (right) homoeostasis, together with their expression values (FPKM) of their mRNA transcripts across libraries derived from individual developmental stages of *Dermanyssus gallinae*. Accession numbers are available in Supplementary Table S6. UP unfed protonymphs, FP fed protonymphs, FD fed deutonymphs. *alas*, 5-aminolevulinate synthase; *pbgs*, porphobilinogen synthase; *hmbs*, hydroxymethylbilane synthase; *uros*, uroporphyrinogen synthase; *urod*, uroporphyrinogen decarboxylase; *cpox*, coproporphyrinogen oxidase; *ppox*, protoporphyrinogen oxidase; *fech*, ferrochelatase; *ho*, haem oxygenase; *fer*, ferritin; *tf*, transferrin; *irp*, iron-responsive protein. **b** The LC/MS analysis of Haem *b* precursors in the naive non-fed stages of *D. gallinae*. Reconstructed chromatograms for the *D. gallinae* samples are shown on top, with reconstructed chromatograms for their analytical standards below. **c** A schematic of haem biosynthesis, which identifies (filled circles) transcripts and metabolites of the pathway. ALA δ-aminolevulinic acid, PBG porphobilinogen, HMB hydroxymethylbilane, URO uroporphyrinogen, CPP coproporphyrinogen, PP protoporphyrin. **d** A photographic image of eggs of *D. gallinae* mites and *Ixodes ricinus* ticks; note the colour difference, indicating the absence/presence of maternal haem deposits.

Apart from being protein-rich, host blood is also a rich source of haem- and non-haem iron. Midgut-specific expression of transcripts that encode proteins implicated in haem or iron biology, e.g. ferrochelatase, ferritin 2 (Fig. 2d, d**’**), supports their importance for the maintenance of metallostasis in the midgut of *D. gallinae* mites. Unlike ticks, which cannot synthesise haem de novo and must take haem from host blood haemoglobin^22^, *D. gallinae* mites clearly displayed the capacity to synthesise haem de novo. We identified transcripts encoding a full enzymatic profile for haem biosynthesis, including the midgut-enriched *ferrochelatase* transcript (Fig. 4a). To further demonstrate the activity of this pathway, we identified in homogenates of non-fed stages of *D. gallinae*, stable intermediate products of haem biosynthesis, either by standards (Fig. 4b) or by accurate mass determination (coproporphyrinogen III; Supplementary Fig. S3a). The final product of the pathway, haem *b*, was identified by comparison with the haemin standard, with [M + 2]^+^ as a diagnostic ion within its mass spectrum (Supplementary Fig. S3b), indicating a fully active haem biosynthetic pathway (Fig. 4c). Additionally, *D. gallinae* mites produce colourless eggs lacking deposits of maternal haem (Fig. 4d), favouring the concept of active haem biosynthesis over somatic distribution and deposition of dietary haem as operated in ticks^22^. This provides another layer of evidence for a difference in the haem biology of ticks^23^ and *D. gallinae* mites, despite both being phylogenetically related obligatory blood-feeders.

Similarly to ticks and other mites^22^, *D. gallinae* mites do not code for haem oxygenase (Fig. 4a), a haem-cleaving enzyme that liberates bioavailable iron from the porphyrin ring. Acquired dietary iron must thus be of non-haem origin. Upon uptake, iron gets sequestered by intracellular iron binders. We identified two *D. gallinae* transcripts encoding large iron-binding ferritin complexes. The transcript of *ferritin 1* (*fer1*) has a conserved iron-responsive element in the 5′UTR region (Supplementary Fig. S4), suggesting its post-translational regulation dependent on iron bioavailability. Additionally, we identified a second *ferritin* transcript (*fer2*) encoding ferritin protein with a predicted signal peptide (DeepLoc: 0.77) at the N-terminus (Supplementary Fig. S4), indicating its role in iron inter-tissue trafficking. Only a single homologue of insect type 2 transferrins (melanotransferrins) was identified in the *D. gallinae* transcriptome (Fig. 4a).

### Comparison of adult and immature stages reveals transcripts involved in vitellogenesis and reproduction

**Fig. 5:**
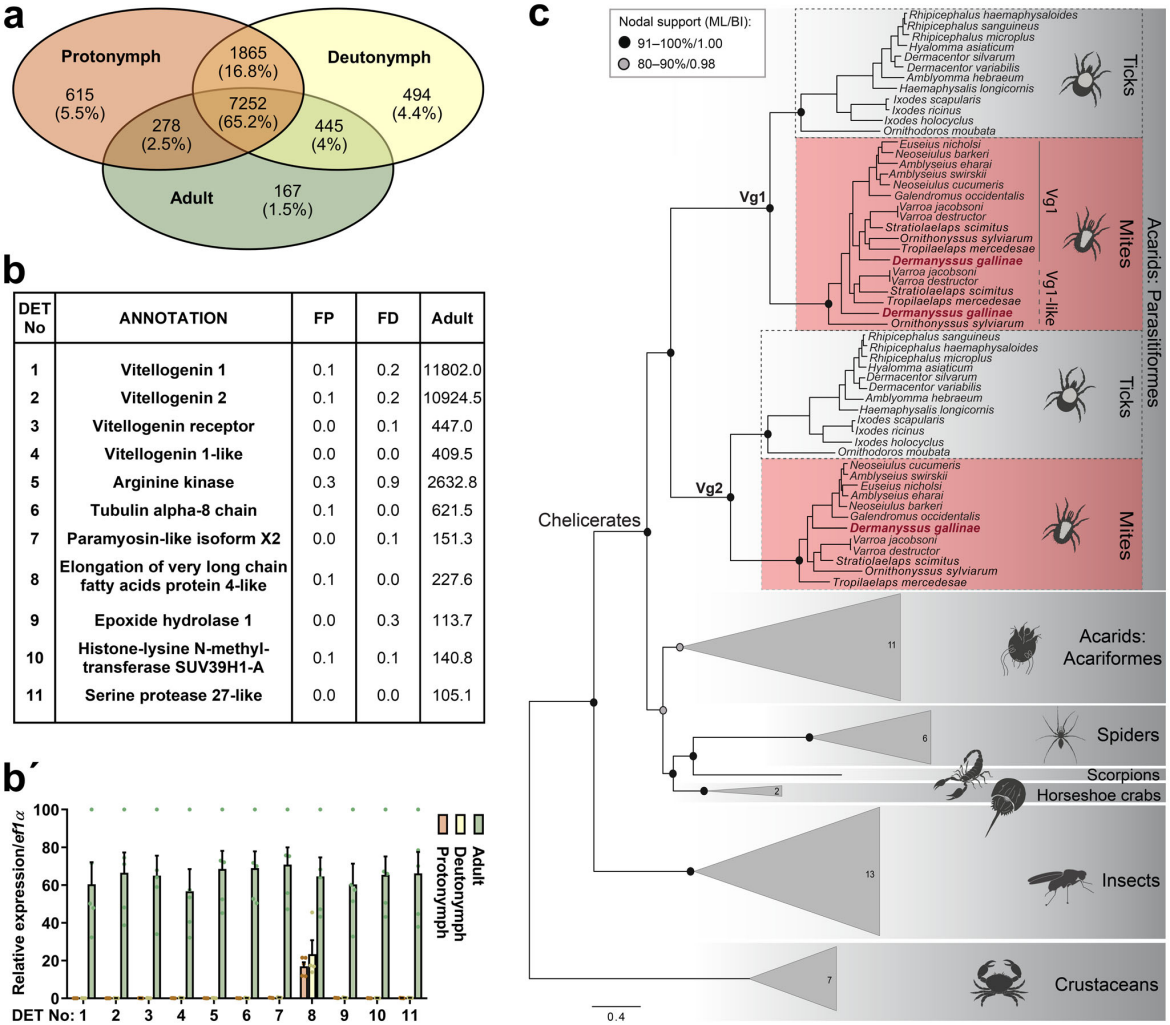
Enriched transcripts in adult mites and phylogenetic reconstruction of arthropod vitellogenins. **a** Venn diagrams show the stage-specific transcriptomic idiosyncracies as well as the transcriptomic core shared across ontogeny. **b** The table shows the top transcripts enriched in transcriptomes of adult mites. These were filtered by >16× FPKM values in the transcriptome of adults over transcriptomes of both mite juvenile stages, protonymphs and deutonymphs; and *E*-value < e^−80^; protein IDs are available as Supplementary Table S7. **b’** RT-qPCR validation of DETs identified by RNA-seq data shown in panel (**b**). Data were obtained from cDNA sets synthesised from three independent RNA isolates of adult and juvenile mites (*n* = 3) and normalised to *elongation factor 1* (*ef1α*). Means and SEMs are shown. For the source data behind the graph, see Supplementary Data S1. FP fed protonymphs, FD fed deutonymphs. **c** Maximum likelihood phylogeny of 94 arthropod vitellogenin amino acid sequences showing the positioning of three *D. gallinae* vitellogenin homologues within the Vg1 and Vg2 lineages. Crustacean vitellogenins were used as an outgroup. Nodal supports at the main nodes are represented by maximum likelihood bootstrap values and Bayesian inference posterior probabilities, respectively. For simplification, the homologues from non-parasitiform taxa were collapsed into triangles; numbers inside the triangle indicate the number of sequences included in each clade. For GenBank accession numbers, see Supplementary Table S8.

We identified 7252 coding sequences (65.2%) shared across developmental stages (Fig. 5a), and these represent the transcriptomic core of *D. gallinae* mites. Indeed, immature stages (protonymphs and deutonymphs) shared more transcripts with each other than any of the immature stages with adults (Fig. 5a). Each stage expressed 4.4–5.5% of unique idiosyncratic transcripts (Fig. 5a). Libraries of adult mites were characterised by highly abundant transcripts encoding proteins that participate in energy maintenance (arginine kinase) and nutrient storage/distribution (vitellogenins and vitellogenin receptor; Fig. 5b). Using protein primary sequences of *I. ricinus* vitellogenin 1 and 2 as tBlastN queries, we identified, within the adult *D. gallinae* transcriptome, two vitellogenin transcripts encoding the homologues of tick vitellogenins^22^, but also an additional vitellogenin transcript (vitellogenin 1-like). All three transcripts clearly displayed enhanced levels within the transcriptomes of adult mites, with virtually no mRNA present in the transcriptomes of juvenile stages (Fig. 5b, b**’**). The additional transcript encoding distinct vitellogenin seems to be unique for Dermanyssoidea mites and is denoted here as Vg1-like (Fig. 5c, Supplementary Table S8). Phylogenetic analysis of arthropod vitellogenins produced several vitellogenin clades reflecting their arthropod taxonomic grouping, i.e., parasitiform ticks and mites, acariform mites, spiders, scorpions, horseshoe crabs, insects, and crustaceans (Fig. 5c). The acarids split into two well-supported clades containing homologues from the Parasitiformes (ML/BI = 95/1.00) and the Acariformes (ML/BI = 84/0.98). Parasitiform sequences clustered within the well-supported Vg1 and Vg2 lineages (both ML/BI = 100/1.00), each comprising two ticks and two to three mite homologues. The *D. gallinae* mite Vg1 (transcript IrSigP-350331_FR2_207-2055, Protein ID: MBD2876257.1), Vg1-like (transcript IrSigP-349783_FR3_1-1870, Protein ID: MBD2876000.1) and Vg2 (Dg-9795_FR3_189-2089, Protein ID: MBD2876256.1) grouped with their respective mite vitellogenin orthologues within the corresponding Vg lineages (Fig. 5c).

To assess whether *D. gallinae* mites convey feeding status through a TOR-mediated transduction cascade, using *Caenorhabditis* homologues as query sequences^24^, we have mined our transcriptomes and identified expression of the TOR-complex system and insulin/IGF signalling^25^ being expressed across developmental stages (Supplementary Fig. S5a). We further evaluated the impact of TOR-mediated signalling on the post-feeding viability of mites, using an ex vivo membrane feeding system^26^, by feeding adult *D. gallinae* mites with chicken blood supplemented with Torin2, an inhibitor of TOR complexes^27^. The resulting data show that Torin2 caused a clear dose-dependent post-blood-feeding lethality at high inhibitor dosage (Supplementary Fig. S5b), indicating a possible post-feeding activity of this pathway in adult mites.

### Cataloguing of ligand-gated ion channels and probing sensitivities to commercial acaricides

**Fig. 6:**
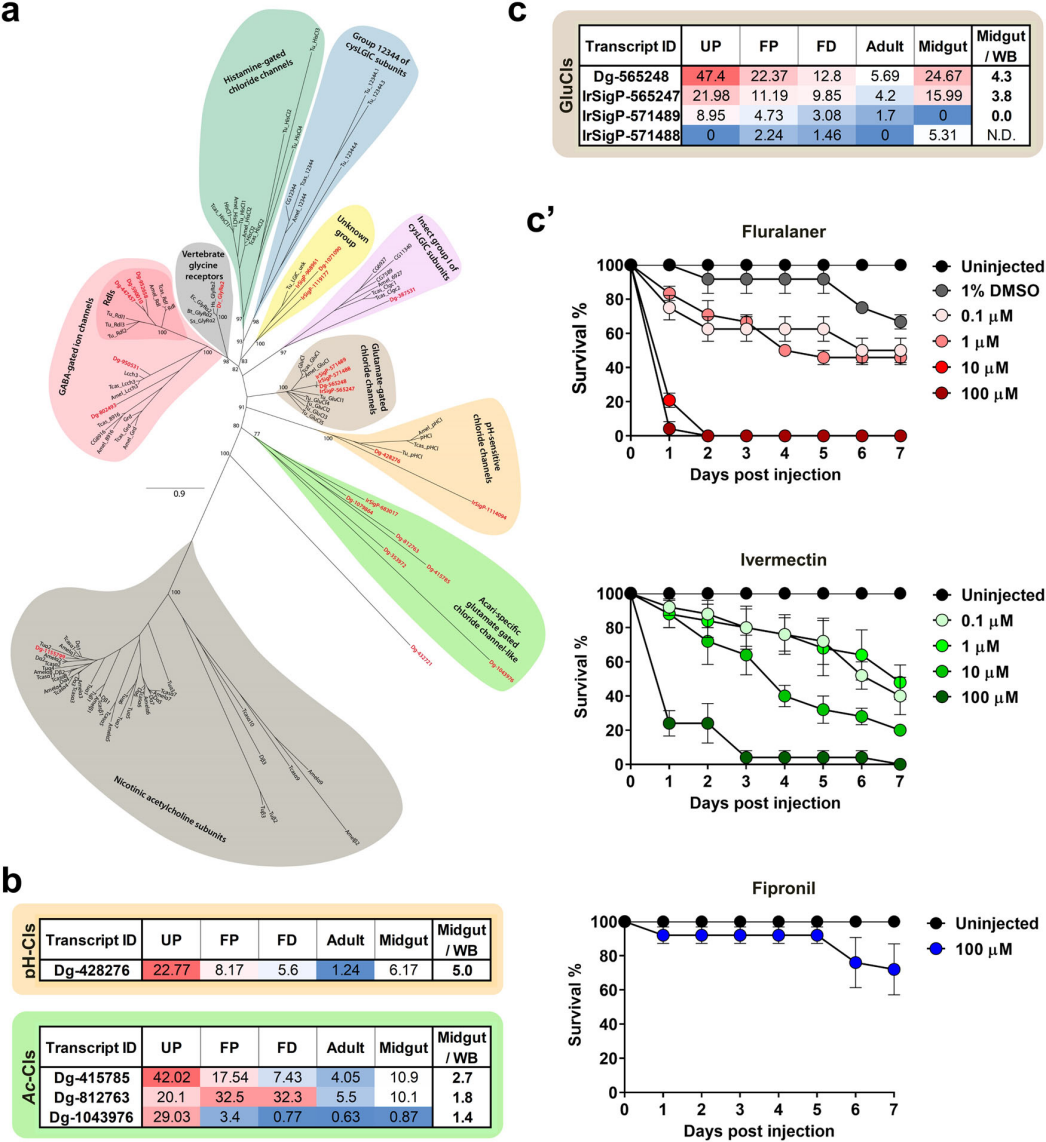
Glutamate-gated chloride channels as acaricidal targets. **a** Maximum likelihood phylogenetic tree of the arthropod and vertebrate cysLGIC subunit protein sequences. The bootstrap supports above 50% are shown at the main nodes. *Dermanyssus gallinae*: Dg and IrSig (indicated in red), *Tetranychus urticae*: Tu, *Drosophila melanogaster* (D or other), *Apis mellifera* (Amel), *Tribolium castaneum* (Tcas), *Homo sapiens* (Hs), *Danio rerio* (Dr), *Bos taurus* (Bt), *Equus caballus* (Ec), *Sus scrofa* (Ss), and *Gallus gallus* (Gg). For accession numbers, see Supplementary Data S1. **b** FPKM values of individual Acari-specific (Ac) and pH-dependent (pH) chloride channels (Cls) and their midgut/whole body (WB) ratio of adult mites. UP unfed protonymphs, FP fed protonymphs, FD fed deutonymphs. **c** FPKM values of individual GluCls and their midgut/whole body ratio of adult mites. **c’** Dose-dependent viability assays after microinjection of GluCls agonists dissolved in DMSO and diluted to 1% of the solvent. Each data point represents a mean, and SEM of *n* = 24 or 25 mites per tested concentration. For the source data behind the graphs, see Supplementary Data S1.

The majority of commercially available acaricides, as well as insecticides, target invertebrate nervous systems, usually by interaction with an allosteric site of ionotropic receptors, also called ligand-gated ion channels (LGICs)^28^. Members of this family have a high degree of primary sequence similarity, forming a ligand-binding site for neurotransmitter molecules and an ion-conducting pore. LGICs can be categorised into four major families^29^, one of which entails a series of validated acaricidal/insecticidal targets, i.e. the pentameric cys-loop family, which shares a highly characteristic motif, a 15-aa cys loop in the protein N-terminus^30,31^. The cys-loop receptors (cysLGICs) are both cation-permeable, such as nicotinic acetylcholine receptors (nAChRs)^32^, serotonin receptors, and also receptors that conduct anions, such as γ-amino butyric acid (GABA)-gated ion channels^33^, glycine receptors, and chloride channels gated by glutamate (GluCls), histamine^34,35^ or zinc^36^. The last two, histamine- and zinc-gated chloride channels, were not identified in the *D. gallinae* transcriptome (Supplementary Data S2). Unlike some pentameric ligand-gated ion channels, homologues of RDL(resistance to dieldrin)-GABA receptors^28^ and GluCls are unique to invertebrates^37^. RDL-GABA and GluCls can be targeted by negative modulators, such as Fipronil, or positive modulators, such as Ivermectin^38^. We have mined our transcriptome (*E-*value < e^−10^, using *Pediculus* sp.^39^ and *Tetranychus* sp.^40^ as queries) and reconstructed a phylogenetic tree of cys-loop receptors, having identified 29 transcript-encoding members of the cys-loop family (Fig. 6a). The *D. gallinae* transcripts clustered with their respective homologues from arthropods and vertebrates into nine clades and in an additional clade that exclusively united the Acari-specific glutamate-gated chloride channel-like proteins. Looking at the midgut/whole body ratio, we filtered out four more transcripts with a midgut-enriched presence of mRNA transcripts (Fig. 6b), one encoding the pH-sensitive chloride channel protein and three encoding Acari-specific chloride channel-like proteins. We next identified four transcripts encoding GluCls, two of which displayed midgut-specific expression (Fig. 6c), but with all transcripts sharing a core of GluCls with the Cys-Loop region and mostly four transmembrane domains (Supplementary Fig. S6). The amino acid sequence analysis of GluCls demonstrated the conservation of most of the amino acid residues required for Ivermectin association with the *C. elegans* GluCl alpha channel^41^ (Supplementary Fig. S6). The assembly of the four GluCl homologues was supported by multiple reads, except for the encoded C-terminus of the IrSigP-571488 transcript, which is composed of uniquely assigned reads (Supplementary Fig. S7). To confirm the sensitivity of *D. gallinae* mites to acaricides targeting GluCl channel(s), as exemplified by Fipronil, Ivermectin, and Fluralaner, we experimentally determined dose-dependent survival after microinjection of the respective compounds into adult mites (Fig. 6c**’**). While Ivermectin and Fluralaner elicited clear lethal effects within a couple of days upon microinjection of a 10 µM solution, Fipronil caused only negligible lethality even when injected at 100 µM (Fig. 6c**’**).

### Mining of immune signal transduction pathways and characterisation of their immune elicitors

**Fig. 7:**
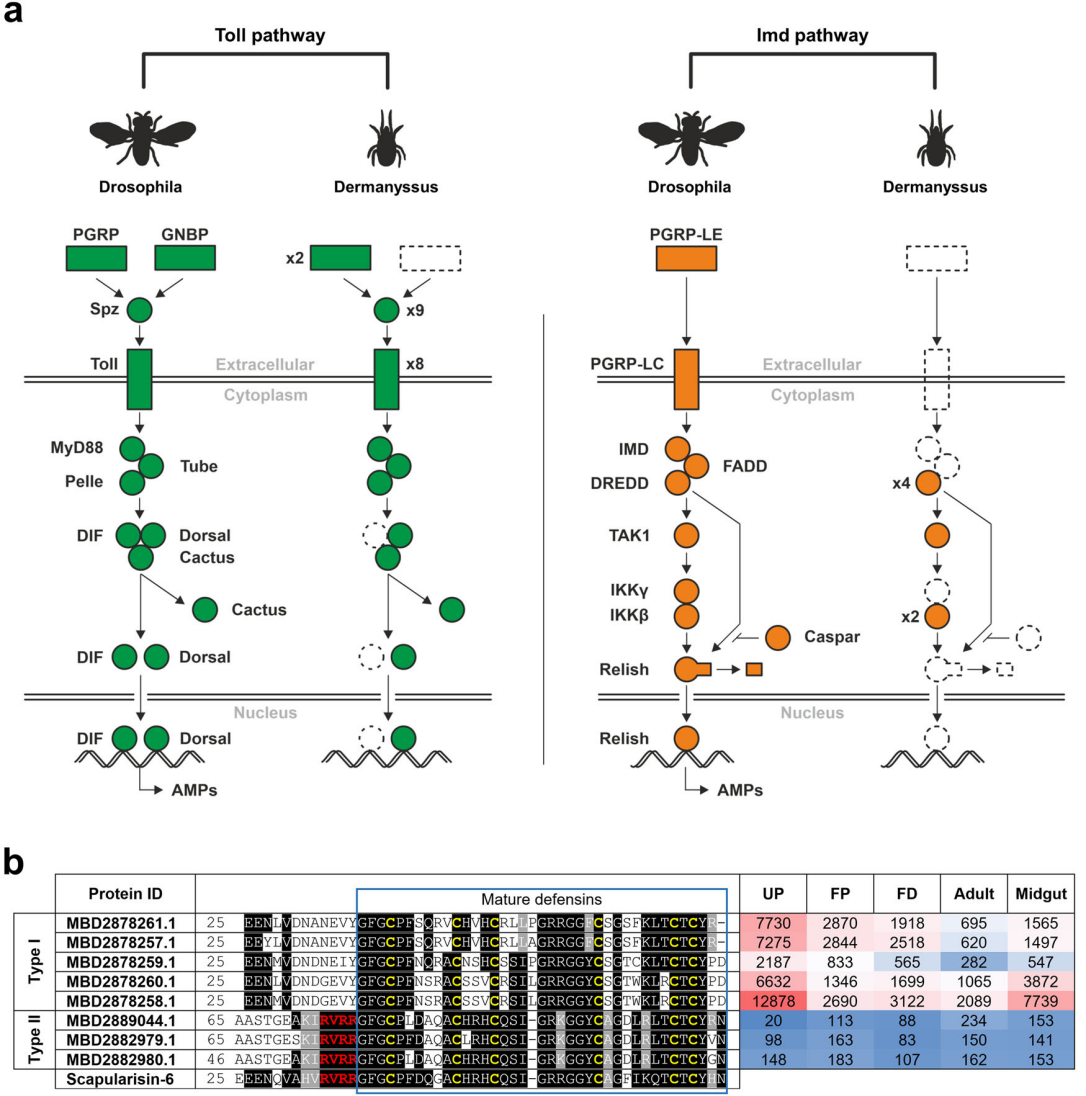
Mining of immune pathways in the *D. gallinae* transcriptome. **a** Protein sequences of *Drosophila* or *Tribolium* Toll and Imd pathways were used to search in our *D. gallinae* translated protein database (Local BLAST, *E*-value 0.1, Matrix BLOSUM62). Conservation of the domains was checked by CD-search (NCBI). Only homologous sequences with similar domain structures and an *E*-value < 10e^−3^ were considered putative homologues. Accession numbers are available as Supplementary Table S9. **b** Multiple sequence alignment of identified *D. gallinae* mature defensins, homology with tick *Ixodes scapularis* scapularisin-6 (GenBank EEC08935) and expression values (FPKM) of defensin transcripts across libraries derived from individual developmental stages of *D. gallinae*. The amino acid residues of the Furin cleavage site are shown in red letters. UP unfed protonymphs, FP fed protonymphs, FD fed deutonymphs.

Innate immunity is highly conserved in metazoans, spanning both vertebrates and invertebrates^42^. The humoral part of invertebrate immunity comprises the sensing of microbial non-self, which is followed by signal transduction, mainly through Toll and Imd signalling pathways that operate, to some extent, independently^43^. In our work, we mined the *D. gallinae* transcriptome and reconstructed a complete Toll pathway, suggesting full functionality in *D. gallinae* (Fig. 7a). The only missing components were Gram-negative bacteria-binding proteins (GNBPs) and transcription factor DIF. Peptidoglycan recognition proteins (PGRPs) were present in two different variants with uncertain classification. Nine Spätzle proteins and eight Toll receptors were identified. The Imd pathway was significantly reduced, as most components were missing, including IMD and Relish. Defensins are probably the most widely spread antimicrobial peptides of 3–5 kDa, broadly distributed in the animal and plant kingdoms^44^ and are effector molecules regulated by both the Toll and IMD pathways^45^. We searched for defensin-like molecules in the *D. gallinae* transcriptome using data from the tick *Ixodes scapularis* genome that encodes multiple defensin-related molecules, divided into two major families: (i) scapularisins, structurally related to the ancient invertebrate-type defensin and (ii) scasins, which are only distantly related to the canonical defensins^46^. While we failed to find any transcript related to the *I. scapularis* scasin (Gen Bank EEC18782), we identified several defensin-like molecules related to the scapularisins with the highest homology displayed to scapularisin-6 (GenBank EEC08935) annotated as defensin-2 in the *I. scapularis* genome (VectorBase ISCW005928). Sequences of eight *D. gallinae* transcripts encoding putative defensins aligned with scapularisin-6 (Fig. 7b) revealed that *D. gallinae* defensins could be divided into two major types: (i) Type I defensins lacking the canonical furin cleavage motif (RVRR)^47^ and (ii) Type II defensins having the furin site conserved. Based on the FPKM values, Type I defensins seem to be much more expressed in all stages (Fig. 7b).

The principal role in cellular and humoral innate immunity of vertebrates, as well as invertebrate metazoan organisms, is played by the complex complement system^48^. The central effector molecules of vertebrate and invertebrate complement systems are proteins belonging to the thioester-containing protein (TEP) family, formerly referred to as proteins of α_2_-macroglobulin superfamily^48–50^. In invertebrates, the TEP family is formed from up to four major phylogenetically distinct groups comprising pan-protease inhibitors of the α2-macroglobulin type (α2M), C3-like complement components (C3), insect-type TEPs (iTEPs), and macroglobulin-complement related (MCRs)^51–53^.

Here, we examined the *D. gallinae* transcriptome with full sequences of nine well-annotated members of the *I. ricinus* TEP family representing all four groups of invertebrate TEPs^52,54^. BlastP mining, and the following phylogenetic analyses with other selected representatives of invertebrate TEPs, revealed that *D. gallinae* expressed only one α2-macroglobulin molecule, which was encoded by at least five splicing variants (DgA2M(sv1–5) (Supplementary Fig. S8) within the protease-sensitive ‘bait-region’. Further, the *D. gallinae* transcriptome contained one molecule clearly belonging to the insect-type TEP (*Dg*TEP), one C3-complement like molecule (*Dg*C3) and three molecules belonging to the group of macroglobulin-complement-related proteins (*Dg*MCR-1,2,3) (Supplementary Fig. S8). *Dg*MCR-3 appears to be phylogenetically quite distant from the other two, *Dg*MCR-1, and *Dg*MCR-2, yet its membership within the MCRs is supported by its stable phylogenetic positioning inside the MCR clade and by the presence of the signature low density lipoprotein receptor domain in the central part of the molecule^52,54^. Taken together, *D. gallinae* possesses representatives of all major groups of invertebrate TEPs, most of which display substantial differences in their expression pattern (Supplementary Fig. S8).

Arthropods^55^, similarly to higher organisms^56^, are armed with an RNA-based mechanism of anti-viral immunity called RNA interference (RNAi). This pathway is functional against both RNA and DNA viruses in insects^57,58^. Both in silico predictions and experimental validation support the presence of a functional RNAi pathway in *D. gallinae*^59^. We confirmed (Supplementary Fig. S9) the in silico analysis^59^ and identified most transcripts encoding RNAi proteinaceous components, with the exception of an R2D2 homologue (Eval cut off < 0.00001; query *Drosophila* R2D2: NP_001285720.1). R2D2, a dsRNA binding protein, is reported to be essential for the loading of siRNAs into effector Ago-RISC complexes^60^. This is in line with a comprehensive phylogenetic analysis that determined the existence of R2D2 homologues only in orders of winged insects (Pterygota) but their absence in non-insect arthropods (ticks, mites, crustaceans)^61^.

### Description of the RNA-virome in *D. gallinae* mites

**Fig. 8:**
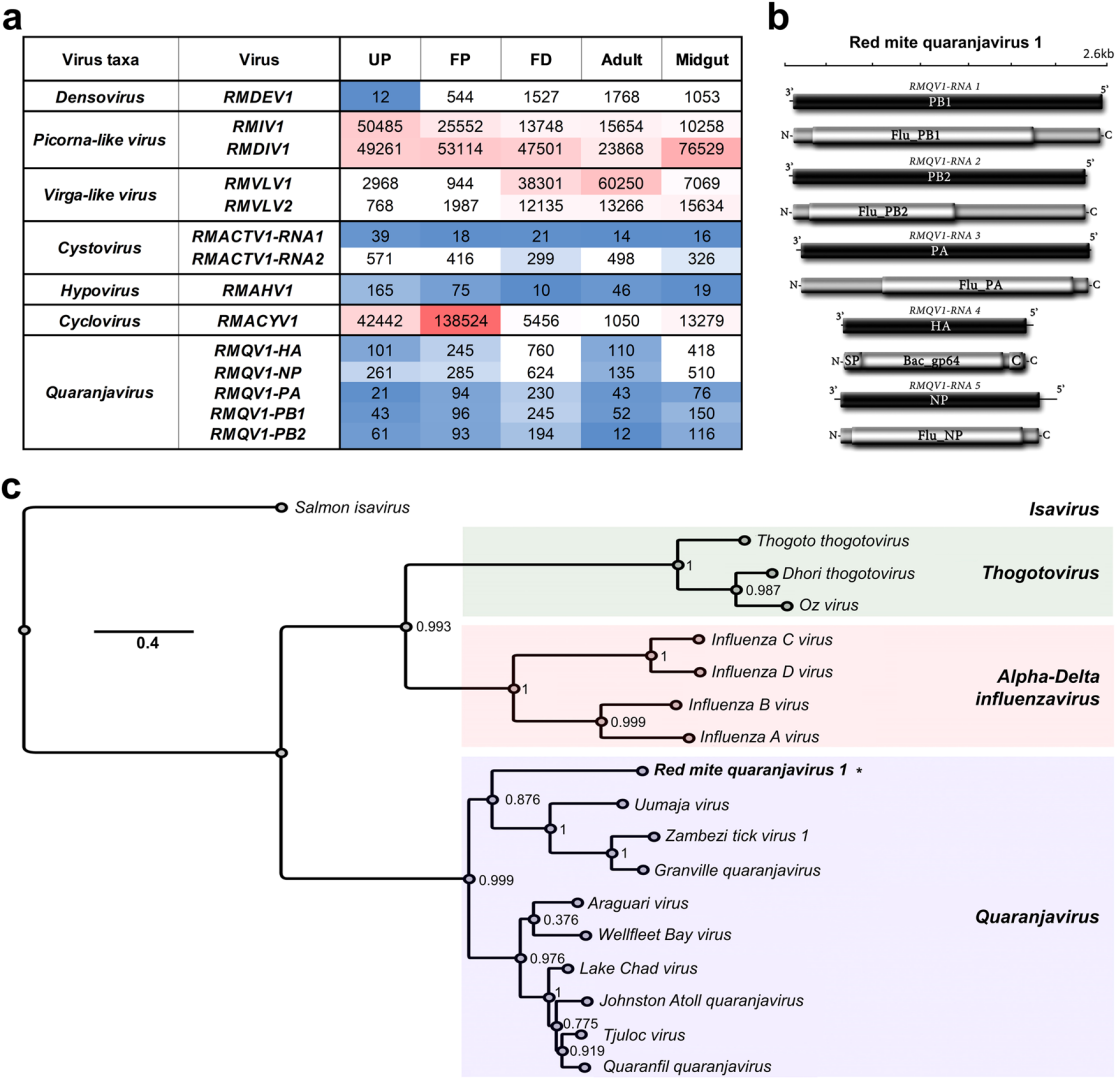
RNA-virome in *D. gallinae* mites. **a** Identified viral segements and their respective gene abundances across libraries derived from individual developmental stages of *D. gallinae*. UP unfed protonymphs, FP fed protonymphs, FD fed deutonymphs. **b** Genome graphs depicting genome segments and predicted gene products of Red mite quaranjavirus 1. Black rectangles indicate ORFs coding sequences, grey rectangles predict proteins and light grey rectangles indicate coordinates of structural or functional domains. Abbreviations are described in the main text. (Right panel) Expression values for identified Red mite quaranjavirus segments. **c** Maximum likelihood phylogenetic tree based on the alignment of predicted PB1 and NP proteins of Red mite quaranjavirus 1 (asterisk) and related viruses. Branch labels represent FastTree support values. The scale bar represents substitutions per site.

As a part of our aim to characterise the complete RNA component of *D. gallinae*, we oriented our search using a standard pipeline of arthropod virus discovery to detect potential viral like sequences in our red mite RNA-seq samples. The presence of viral sequences in libraries of UP (not exposed to host blood feeding) or dissected midguts (dissected interior tissue) demonstrated a genuine *D. gallinae* virome, consisting of red mite picorna-like virus, red mite densovirus, red mite virga-like virus, red mite associated cyclovirus, red mite associated hypovirus, and red mite associated cystovirus (Fig. 8a). Additionally, several putative transcripts were identified with significant homology to viral proteins, such as that of a 1.5 kb transcript with resemblance to the hemagglutinin protein (HA) of Wellfleet Bay virus (WFBV, 33.85% identity, *E*-value 9e^−96^) (Fig. 8a). In additional tblastN searches using all available quaranjavirus proteins as queries, we detected four further transcripts resembling all the typical core genome segments of orthomyxoviruses. This included a sequence homologous to a 1.8 kb transcript of the nucleoprotein (NP) of *Quaranfil quaranjavirus* (QRFV, identity 30.20%, *E*-value 6e^−57^). Besides these structural proteins, we found three transcripts ranging from 2.4 kb to 2.5 kb with best hits to the multipartite RNA-dependent RNA polymerase subunit proteins PB1 (Tjuloc virus, identity 50%, *E*-value 0), PB2 (QRFV, identity 28.90%, *E*-value 9e^−94^) and PA (Tjuloc virus, identity 50%, *E*-value 0). These transcripts were polished and curated using the filtered reads of each library to generate consensus sequences of each genome segment, supported by a mean coverage ranging from 13.5× to 47.5×. The putative genome segments of the expected size were further annotated and, as anticipated, each presented a single ORF in their complementary RNA+ strand coding for the structural and functional proteins of a typical quaranjavirus, flanked by untranslated regions (Fig. 8b). The translated polymerase subunit proteins PB1, PB2 and PA presented with conserved domains Flu_PB1 (pfam00602, *E*-value 3.62e^−60^), Flu_PB2 (5D98_F, *E*-value 8.2e^−121^), and Flu_PA (4WRT_A, *E*-value 4.9e^−97^), respectively. The NP showed a Flu_NP domain (3TJ0_B, *E*-value 7.5e^−81^), and the putative surface HA protein harboured a typical Baculovirus gp64 envelope glycoprotein domain (Bac_gp64, *E*-value 4.64e^−48^), a signal peptide (SP) at the N terminus for transmembrane passage and a C terminal domain. We then surveyed the diverse libraries of *D. gallinae* to assess the presence of those genome segments that were detected in all libraries following symmetrical expression patterns, based on FPKMs and supporting the possibility that the segments corresponded to the same virus (Fig. 8a). Expectedly, expression values of the structural segments were higher than the nonstructural ones in each library, which is indirect evidence of active infection (Fig. 8a). Based on these genomic data, genetic distance, functional and structural annotation, and expression profiles, we suggest that these genome segments correspond to a novel virus; a putative member of a new species within the genus *Quaranjavirus* that we tentatively named Red mite quaranjavirus 1 (RMQV1). To entertain this hypothesis, we generated evolutionary insights based on the phylogenetic analysis of PB1 and NP proteins of RMQV1 and diverse orthomyxoviruses. The resulting tree indicated clearly that RMQV1 clustered within the quaranjavirus clade (Fig. 8c). In summary, a diverse and complex red mite virome was detected and characterised for *D. gallinae*, including novel viral species of several families of viruses, having potentially important effects on the biology and health of their hosts. Future studies aimed at determining the epidemiological, ecological and veterinary effects of these viruses are warranted.

## Discussion

Blood feeding has evolved several times in Acari^62^. The blood-feeding arthropods display a remarkable capacity for rapid proteolysis of host blood, which is a very rich source of proteins (90% dry weight). The haemoglobinolytic apparatus in their digestive tracts was suggested to be shared among phylogenetically related blood-feeding parasites and was highlighted as promising anti-parasite targets^63^. Although ticks and dermanyssoid mites belong to the same order Acarina (class Arachnida), and both represent blood-feeding ectoparasites, the data obtained here support the hypothesis of the independent appearance of blood-feeding. This is substantiated by their clear differences in digestive proteolytic machinery. While both hard (*I. ricinus*) and soft ticks (*Ornithodoros moubata*) rely on cathepsin B as the major haemoglobinolytic peptidase^64^, which also holds true for Acariform mites^65^, we did not identify any cathepsin B-like or cathepsin C-like protease-encoding transcripts in transcriptomes of *D. gallinae* (Mesostigmata mites). The blood digesting proteolytic apparatus of *D. gallinae* mites thus seems to rely mainly on acidic lysosomal proteases (legumain, cathepsin L, and cathepsin D), which account for the majority of all protease-encoding reads in the adult *D. gallinae* midgut. This finding confirms the earlier observation, showing a clear capacity of *D. gallinae* homogenate to digest haemoglobin at an acidic pH, with activity being inhibited by E-64 and pepstatin A, indicating the participation of cysteine and aspartic proteases^66^. Tick digestive cathepsin L (*Ir*CL1) is known to display clear haemoglobinolytic and albuminolytic activities, with a peak at pH 3.5, indicating its acidic mode of operation within the endolysosomal vesicles of tick gut cells^67^. It is, therefore, reasonable to speculate that the engagement of *D. gallinae* Cathepsin L proteases might serve in the analogous biological process of host blood protein digestion, possibly also within intracellular acid-lysosomes. Additionally, transcripts for Cathepsin L5 and Legumain 4 show a clear midgut-specific up-regulation with blood-feeding similarly to ticks^68^, indicating their direct involvement in digestion of host blood proteins. However, a detailed mapping of proteolytic activities present in *D. gallinae* midguts, based on specific substrates and inhibitors, remains an experimental challenge for upcoming research in the field. In summary, after less-than-an-hour of on-host feeding^69^, during which time mites increased their body weight by ~ ten fold^70^, rapid off-host digestion followed. In the close relative dermanyssoid mite *Ornithonyssus sylviarum*, about half of the imbibed erythrocyte content was digested within 4–8 h^71^. Similarly to ticks and acariformes mites^72^, parasitiformes blood-feeding mites (*D. gallinae*)^66^ appear to digest host blood intracellularly, using a multi-enzyme complex of mainly acidic endolysosomal proteolytic enzymes, yet they lack any cathepsin B and C homologues.

It is now clear that *D. gallinae* mites code for full haem biosynthesis. Yet, the heterogeneity of expression profiles of individual transcripts encoding haem biosynthetic enzymes does not fully identify at what developmental stage the biosynthetic pathway might be active, showing very little of a developmental stage- or feeding-status-dependent expression pattern. Transcript encoding ferrochelatese, the final haem-biosynthetic enzyme, was shown to be significantly enriched in midguts, similarly to partially-fed ticks^73^, raising a question of the need for this enzyme in this space and time since the tissue is flooded by cupious amounts of haem. Given the lack of haem deposits in *D. gallinae* eggs, maternally acquired haem probably does not play a key role in embryogenesis and reproduction, as it does in ticks^22^. This is also clearly evidenced by the lack of colour in deposited eggs, which contain estimated subpicomol levels of haem *b* per mg of eggs-rich sample; this contrasts starkly with about a nanomol of haem *b* deposited in the eggs of ticks^22^.

Similarly to ticks^74,75^, *D. gallinae* appears to contain two separate ferritin genes that encode cytosolic and secreted ferritins. Expression of transcripts encoding both of these ferritins in *D. gallinae* indicates their key role in iron homoeostasis in their midguts. We assume that ferritin 1, possessing the iron-responsive element in its 5′ UTR and lacking the signal peptide, functions as an intracellular iron storage protein. The secretory ferritin 2, in contrast, has a clear N-terminal signal sequence, suggesting its role in iron export from the midgut and thus facilitating the somatic distribution of midgut-acquired iron similar to ticks and insects^75,76^. Both *D. gallinae* ferritins possess a conserved ferroxidase di-iron centre (facilitating rapid Fe^2+^ to Fe^3+^ oxidation), as well as a ferrihydrite nucleation centre (promoting nucleation and storage of Fe^3+^); these represent signature motifs for Heavy-chain (H) and Light-chain (L) ferritins, respectively. Thus *D. gallinae* ferritins are apparently hybrids of H- and L-types, similarly as experimentally demonstrated for the crustacean ferritin^77^ and reported for tick ferritins 1 and 2^78^. RNAi studies performed by others demonstrated that both ferritins were essential for the survival and reproduction of *D. gallinae* mites^79^. Secretory ferritin (denoted as Dg-Fer1 in that study) has also been shown to be a potent target conferring antibody-mediated protection against *D. gallinae* mites in hens when used as a recombinant antigen^79^. The dietary source of iron, loading both cytosolic and secretory ferritin, is not clear. However, in *D. gallinae*, given the absence of genetic coding for haem oxygenase, an enzyme liberating iron from the porphyrin ring of haem, the bioavailable pool of iron is likely acquired from host blood transferrin, as experimentally determined for ticks^22,80^.

The immune sensing and signalling Toll and IMD pathways, as well as immune elicitors, were reconstructed here based on the presence/absence of known homologues. While we could mine the full Toll pathway, the Imd pathway was significantly reduced, with key components missing, such as IMD and Relish. The retained members identified in *D. gallinae* mites, which are also integrated into the IMD pathway of insects, were Caspase (DREDD, Cysteine-dependent ASPartyl-specific proteASE) and Serine/Threonine Kinase (TAK1 and IKKβ). The absence (not identified in our transcriptome, nor in the genome with an Eval cut off = 0.0001) of a critical component of the pathway, transcription factor Relish, indicated the non-functionality of the IMD pathway in *D. gallinae* mites, similar to the phylogenetically related mites, *Varroa* (Parasitiformes; Eval cut off < 10e^−3^) and *Metaseiulus* (Acariformes)^81^. Mining of the *D. gallinae* transcriptome for components of the primordial complement system, represented by thioester-containing proteins, revealed that this mite, similarly to other chelicerates such as ticks, spiders, and even the horseshoe crab^51,52,82,83^, possesses all major TEP groups, namely α_2_-macroglobulin, C3-like complement component, insect-type TEP, and macroglobulin-complement related (MCRs). However, some of these groups were apparently lost during evolution, for example, C3-like molecules from Crustaceans and Hexapods or α_2_Ms from some insect lineages such as fruit flies or mosquitoes^51^. The single α_2_M found in the *D. gallinae* transcriptome is diversified within the “bait region” by alternative splicing, presumably to extend the portfolio of target proteases inhibited by the mechanism of molecular entrapment^84^, as previously reported for tick α_2_Ms^85,86^.

Apart from silicone dioxide dusting, chemically synthesised anti-parasitics are used in commercial egg-laying to control populations of *D. gallinae* mites. Most of the commercially successful acaricides target ion-gated chloride channels, leading to the limitation or elimination of *D. gallinae* mites in poultry houses^87^. Using micro-injection of selected commercial acaricides suspended in DMSO and subsequent 100× dilution with sterile PBS, we assessed the dose-dependent survival of *D. gallinae* mites. While we could see clear time- and dose-dependent lethality of mites post micro-injection of Fluralaner and Ivermectin, similarly to previous studies exploiting a different route of administration^87,88^, we did not observe the acaricidal effect of Fipronil administered through micro-injection. This observation may be in line with previously reported lower and heterogenous sensitivity of *D. gallinae* mites to topical exposure to Fipronil solution^89^. While we have no knowledge of Acari-specific chloride channels, an increasing body of work is available on pH-sensitive chloride channels in other invertebrates. Interestingly, the pH-sensitive chloride channel from *Bombyx mori* is sensitive to Ivermectin, but non-responsive to Fipronil^90^, which is reminiscent of our data from viability assays upon microinjection of agonists. This may suggest that pH-dependent chloride channels could be key targets for channel agonists like Ivermectin in eliciting acaricidal activity.

## Conclusion

*D. gallinae* is a global and highly debilitating poultry pest. With recent seminal foundations^17,26,91^ for its detailed molecular understanding, novel acaricidal targets, possibly inherent in blood-feeding mites, might be identified and validated. Here, we have sequenced and assembled new *D. gallinae* transcriptomes, comparing blood-fed and unfed mites, as well as whole bodies and micro-dissected midguts. These represent key informative datasets enabling insights into molecular adaptions of this ectoparasite to its obligatory blood-feeding lifestyle. It also provides us with a catalogue of potential targets suitable for target-based acaricide development. The sequences of assembled contigs, annotations, and respective expression values are available through a user-friendly one-piece hyper-linked Excel sheet (see Data availability). Unlike previous studies, our RNA-seq-based work is complemented by viability assays performed through the artificial membrane feeding platform, haemocoel microinjection, and also by mass spectrometry metabolite identification. Finally, we have identified the *D. gallinae*-specific RNA virome and have highlighted viruses of possible concern. This work presents an integrative assessment of assembled and curated Illumina-originating data, describing the molecular traits inherently linked to the blood-feeding lifestyle of *D. gallinae*, and reveals its emerging role as a reservoir of new viral variants.

## Materials and methods

### Collection of mites, RNA extraction, and library preparation

Mites were collected by brushing cages of egg-producing hens in the International Poultry Testing Ustrasice (MTD Ustrasice, Czech Republic). Mites were briefly anaesthetised with CO_2_ using a FlyPad (Flystuff.com) and were separated into three developmental stages: protonymphs, deutonymphs, and adults. Protonymphs were further separated into unfed and blood-fed mites. Midguts were micro-dissected from the adult stages. Mites were placed on a double-sticky tape, ventral side down, decapitated with a razor, and midguts were pulled out into a drop of diethylpyrocarbonate (DEPC)-treated phosphate-buffered saline (PBS). Whole mites of different developmental stages (each *n* = 30) were homogenised with an eppi pestle, and midguts (*n* = 40) were homogenised using a 29 G insulin syringe needle. Total RNA was extracted from the homogenates with a NucleoSpin RNA kit (Macherey Nagel) and eluted into RNase-free water, with a yield of 2–7 µg of total RNA. An Agilent 2100 BioAnalyser evaluated the quality of RNA, with RIN values ≥ 9. A non-stranded cDNA library was prepared by NEBNext® Ultra™ RNA Library Prep Kit for Illumina® and sequenced on NovaSeq600 by Novagene Co., Ltd. as 150 bp paired-end reads.

### Transcriptome assembly, data deposition, filtering of outputs

Transcriptome assembly and coding sequence extraction was carried out as described previously^92^. Briefly, reads were stripped of their contaminating primers, and bases with equal values < 20 were trimmed using the programme Trim Galore (Krueger F. Trim Galore: a wrapper tool around Cutadapt and FastQC. Trim Galore. 2012). Clean reads were assembled using the Abyss^93^ and Trinity^94^ assemblers. These assemblies were merged using a parallelised pipeline of blastn and cap3 assembler^95^ as described previously^96^. We extracted all open reading frames larger than 200 nucleotides, and those matching known proteins or having a signal peptide were retained. The resulting peptide and coding sequences were mapped to a hyperlinked spreadsheet, including blastp and rpsblast matches to several databases, as well as an indication of the signal peptide^97^, transmembrane domains^98^ and O-galactosylation sites^99^. Transcripts assigned to a given developmental stage were filtered by a 16-fold change factor, i.e. to denote a transcript “adult-specific”, its FPKM values were >16 fold over its FPKM values in the transcriptome of FP, and at the same time, in the transcriptome of fed deutonymphs (see “Data availability”). In order to list transcripts enriched in the transcriptome of fed over UP, only transcripts with clear annotations (*E*-value < e^−100^), coverage > 10%, and FPKM in FP > 5 were considered. The transcripts were then listed according to the highest fed/unfed ratios.

### cDNA synthesis and RT-qPCR

We prepared total RNA samples, as above, in 3–5 independent biological replicates. Thirty mites (whole bodies) or midguts from forty adult mites were used for RNA extractions, yielding around 1 μg of RNA per extraction, with the exception of samples of adults (whole bodies), which yielded around 8 μg of RNA per extraction. Single-stranded cDNA was reverse-transcribed from 0.2 µg of total RNA using the First Strand cDNA Synthesis Kit (Roche) with oligo(dT)18 primers. For RT-qPCR, cDNA was diluted 10 times in nuclease-free water, with 10 pmol of each primer, and run in 10 μL reaction in 384-well plates (MicroAmp™ Optical 384-Well Reaction Plate, Thermo Fisher Scientific, USA), using FastStart Universal SYBR Green Master (Rox) (Roche Life Sciences), in QuantStudio™ 6 Flex Real-Time PCR system (Thermo Fisher Scientific, USA). Primers used in this study are listed in Supplementary Tables S3, S4, and S7.

### Phylogenetic analyses

The dataset used for the phylogenetic analyses of arthropod vitellogenins consisted of 67 amino acid sequences representing homologues from ticks, mites, acarids, spiders, scorpions, horseshoe crabs, insects and crustaceans, with the latter being an outgroup (Supplementary Table S8). The vitellogenin sequences were retrieved as GenBank annotated entries or were mined from the genome/ transcriptome assemblies available in GenBank using the tBlastN algorithm and *E*-value cutoff < 10^−5^. The structurally similar haem-lipo-glycoproteins of ticks (e.g., GenBank: ABK40086 and ACF35055) and their related tick sequences annotated in GenBank as vitellogenins (e.g., GenBank: AXP34688, BAJ21514, AXP34687, XP_029826448) were not included in the analysis because these proteins are not true vitellogenin homologues. Vitellogenin sequences were aligned in MAFFT (v 7.017)^100^ implemented in Geneious Prime v2019.0.4^101^ using an automatic selection of the alignment strategy and default parameters for the gap opening penalty (1.53) and the offset value (0.123). We trimmed non-homologous regions of the alignment, using GBlocks v0.91b^102^ under default parameters except with minimum block length set to 5 and with half the gapped positions allowed, so the final alignment comprised 3578 amino acid positions comprising the three structural domains typical for vitellogenins (i.e., the vitellogenin N-terminal region, the domain of unknown function [DUF1943], and von Willebrand factor type D domain). The phylogenetic tree was reconstructed by the maximum likelihood (ML) method in IQ-TREE v1.6.12^103^ using the LG + F + R6 protein model selected by ModelFinder^104^. Bootstraps were based on 1000 replicates. Bayesian inference (BI) analysis was performed in MrBayes v3.2.7a^105^ implemented in CIPRES Science Gateway v3.3^106^ using four simultaneous MCMC chains sampled at intervals of 100 trees and posterior probabilities estimated from 2 million generations. The WAG + F + G4 model of evolution was selected by ModelFinder. The burn-in period represented 10% of all generations. The tree was visualised in Geneious Prime v2019.0.4 and graphically modified in Adobe Illustrator CS5.

For the phylogenetic analysis of the cys-loop ligand-gated ion channel gene family, we used a dataset consisting of 121 amino acid sequences that corresponded to the dataset of Dermauw et al.^40^ and were enriched for the homologues from *D. gallinae* (Supplementary Data S1). The sequences retrieved from GenBank annotated entries were aligned and treated as described above for the vitellogenin dataset. The final alignment comprised 502 amino acid positions. The ML-based phylogenetic tree and its nodal supports were calculated using the LG + I + G4 protein model as described for the vitellogenins.

For the phylogenetic analysis of the thioester-containing protein (TEP) family, the dataset contained 29 amino acid sequences of *D. gallinae* TEPs and its invertebrate homologues from the fruit fly *D. melanogaster*, the hard tick *I. ricinus*, and the horseshoe crab *L. polyphemus* (Supplementary Fig. S8). The sequences were aligned and treated as described above for the vitellogenin dataset. Final alignment of full amino acid sequences (~1500 residues) comprised 2737 amino acid positions. The ML-based phylogenetic tree and its nodal supports were calculated using the BLOSUM62 + G protein model as described for the vitellogenins.

### Metabolomic analysis of haem *b* and its biosynthetic intermediates

For metabolomic analysis, mites collected in a poultry house by brushing were stored in a 1 L bottle with a paper lid at 21 °C in the dark. The next day, a mixture of eggs, larvae, and UP was sorted out as described above. Then the sample of unfed stages (to prevent detection of haem intermediates of host origin), with a predominant fraction of UP from 21.4 mg mites, was extracted with 150 µL of a cold extraction medium, MeOH:ACN:H_2_O (2:2:1 v/v/v), containing an internal standard, 4-fluorophenylalanine (100 µL of 0.5 ng/µL). The sample was then homogenised using a Tissue Lyser II (Qiagen, Prague, Czech Republic) at 50 Hz, 0 °C for 5 min. The mixture was then centrifuged at 7000 RPM and 5 °C for 10 min, and the supernatant was filtered through a 0.2 μm PVDF mini-spin filter (HPST, Prague, Czech Republic) at 8000 RPM and 5 °C for 10 min. Finally, a 50 µL aliquot of the supernatant was used for liquid chromatographic–high-resolution mass spectrometric analysis (LC–HRMS). The LC–HRMS methods were described in detail earlier^107,108^. Briefly: A Q Exactive Plus Orbitrap mass spectrometer coupled with a Dionex Ultimate 3000 liquid chromatograph (all Thermo Fisher Scientific, San Jose, CA, USA) was used for profiling intermediates of haem biosynthesis. Metabolites were separated on a 150 mm × 4.6 mm i.d., 5 μm, SeQuant ZIC-pHILIC (Merck KGaA, Darmstadt, Germany) with a mobile phase flow rate of 450 μL/min, an injection volume of 5 μL, and a column temperature of 35 °C. The mobile phase was: A = acetonitrile, B = 20 mmol/L aqueous ammonium carbonate (pH = 9.2; adjusted with NH_4_OH); gradient: 0 min, 20% B; 20 min, 80% B; 20.1 min, 95% B; 23.3 min, 95% B; 23.4 min, 20% B; 30.0 min 20% B. The Q-Exactive settings were: Mass range 70–1000 Daltons; 70,000 resolution (m/z 200; 3 × 106 Automatic Gain Control (AGC) target and maximum ion injection time (IT) 100 ms; electrospray operated in positive mod: 3000 kV spray voltage, 350 °C capillary temperature, sheath gas at 60 au, aux gas at 20 au, spare gas at 1 au, probe temperature 350 °C and S-Lens level at 60 au. Data were processed using XcaliburTM software, version 4.0 (Thermo Fisher Scientific, San Jose, CA, USA). Compounds used in the study: Deionized water was prepared using a Direct Q 3UV purification system (Merck, Prague, Czech Republic). Methanol and acetonitrile (OptimaTM grade) were purchased from Fisher Scientific (Pardubice, Czech Republic); ammonium carbonate, 25% ammonia solution, 4-fluorophenylalanine, 5-aminolevulinate, haemin, porphobilinogen and protoporphyrin IX from Merck (Prague, Czech Republic). The amount of haem *b* was estimated based on the peak area of the standard.

### Ex vivo artificial membrane feeding

Mites were collected and stored as described above. After 14 days, vital individuals were selected and used for ex vivo feeding experiments. The feeding unit was a plastic cylinder (17 mm in diameter, 70 mm in length) horizontally divided into two equal chambers by a membrane (silicone-impregnated Goldbeater’s skin). The upper chamber contained 2 mL of fresh, defibrinated (by mixing with 4 mm glass beads) chicken blood supplemented with the inhibitor Torin2 (Sigma-Aldrich, catalogue number SML1224). The lower chamber of the membrane feeding device contained the mites. The feeding unit was placed in the dark in an incubator set at 41 °C. After 5 h, fed individuals were collected and stored in 1.5 mL microcentrifuge tubes (10 individuals in each) in an incubator set at 21 °C. Mite viability and vitality were monitored every 24 h for 7 days. Torin2 was dissolved in DMSO and further diluted to final concentrations: 500, 250, 100, 10, and 1 µM. DMSO stocks were diluted in the blood meal to 1% v/v DMSO in the blood meals.

### Microinjection

Mites were collected as described above, and freshly fed adult females were sorted. Mites were immobilised on an adhesive tape, and a volume of 13.8 nL of tested compound (≤1% v/v DMSO) was microinjected into the mite’s haemocoel (Drummond, USA). After 10 min, injected mites were collected into 1.5 mL microcentrifuge tubes (5 individuals in 5 eppi tubes or 8 individuals in 3 eppi tubes for each concentration) and stored in the incubator set at 21 °C. The viability and vitality of the mites were monitored every 24 h for 7 days. Compounds used in the study: Fipronil (Sigma-Aldrich Supelco, catalogue number 16785), Fluralaner (Cayman Chemical, item number 22061), Ivermectin (Sigma-Aldrich, catalogue number I8898).

### Virus RNA identification and analyses

Virus discovery, detection and characterisation were implemented as described elsewhere^109^. In brief, we used transcriptome assemblies generated in this work as the input for virus discovery. The complete NR release of viral protein sequences was retrieved from https://www.ncbi.nlm.nih.gov/protein/?term=txid10239[Organism:exp]. The integrated Red mite RNA assembly was assessed by multiple tBlastN searches (max *E*-value = 1 × 10^−5^) using, as a probe, the complete predicted non-redundant viral proteins in a local server. Significant homologies were screened manually, and redundant contigs were discarded. Potential virus-like sequences were curated by iterative mapping of reads using Bowtie 2 v2.4.4^110^. Open reading frames (ORF) were predicted by ORFfinder. Predicted proteins were subjected to BlastP searches at NCBI and to domain-based Blast searches against the Conserved Domain Database (CDD) v3.19 implemented in https://www.ncbi.nlm.nih.gov/Structure/cdd/cdd.shtml and supplemented with SMART http://smart.embl-heidelberg.de/, Pfam http://pfam.xfam.org/, PROSITE http://prosite.expasy.org/ and HHPred https://toolkit.tuebingen.mpg.de/tools/hhpred to characterize more divergent functional domains. Signal and membrane peptides were assessed with SignalP v4.1^111^. Viral RNA levels were calculated with Cufflinks http://cole-trapnell-lab.github.io/cufflinks/ or alternatively with the Geneious suite 8.1.9 (Biomatters Inc.) as Fragments Per Kilobase of virus transcript per million mapped reads (FPKM). Evolutionary insights were resolved by MAFTT (v 7.310)^100^ alignments of amino acid sequences of the predicted viral polymerases or capsid proteins, which were used for phylogenetic analyses based on FastTree approximately maximum-likelihood phylogenetic trees http://www.microbesonline.org/fasttree/ with standard parameters. Support for individual nodes was assessed using an approximate likelihood ratio test with the Shimodaira–Hasegawa-like procedure. Tree topology, support values and substitutions per site were based on 1000 tree resamples. Most sequence analysis results were integrated and visualised using the Geneious suite 8.1.9 (Biomatters Inc.). Virus genome diagrams were designed using Adobe Photoshop C3 version 10.

### Reporting summary

Further information on research design is available in the Nature Portfolio Reporting Summary linked to this article.

## Supplementary information

Supplementary Information

Description of Additional Supplementary Files

Suplementary Data S1

Suplementary Data S2

Reporting Summary

## Data Availability

The data are available as BioProject PRJNA597301, including nt and aa fasta files, and a hyperlinked Excel sheet is available to download at https://proj-bip-prod-publicread.s3.amazonaws.com/transcriptome/Dermanyssus_gallinae/Derm_gallinae.zip. Viral sequences were deposited at the NCBI with the following GenBank accession numbers: RMACTV1-L,ON160022;RMACTV1-S,ON160023;RMQV1-HA,ON160024;RMQV1-NP,ON160025; RMQV1-PA,ON160026;RMQV1-PB1,ON160027;RMQV1-PB2,ON160028;RMDIV1,ON160029; RMIV1, ON160030;RMDEV1,ON160031;RMVLV1,ON160032;RMVLV2,ON160033;RMACYV1,ON160034;RMAHV1,ON160035. Raw data used for generating Fig. 4b were deposited at the Figshare repository with the following DOIs: 10.6084/m9.figshare.22658317.v1, 10.6084/m9.figshare.22658338.v1, 10.6084/m9.figshare.22658386.v1, 10.6084/m9.figshare.22658458.v1.
