# Supplementary material for: Blood-feeding adaptations and virome assessment of the poultry red mite Dermanyssus gallinae guided by RNA-seq
Source: Commun Biol. 2023 May 13;6:517. doi: 10.1038/s42003-023-04907-x (PMC10183022; doi:10.1038/s42003-023-04907-x)
Supplement: Supplementary file 1 — Supplementary Information [file 42003_2023_4907_MOESM1_ESM.pdf]

Supplementary information (Figures and Tables)

Supplementary Fig. S1:

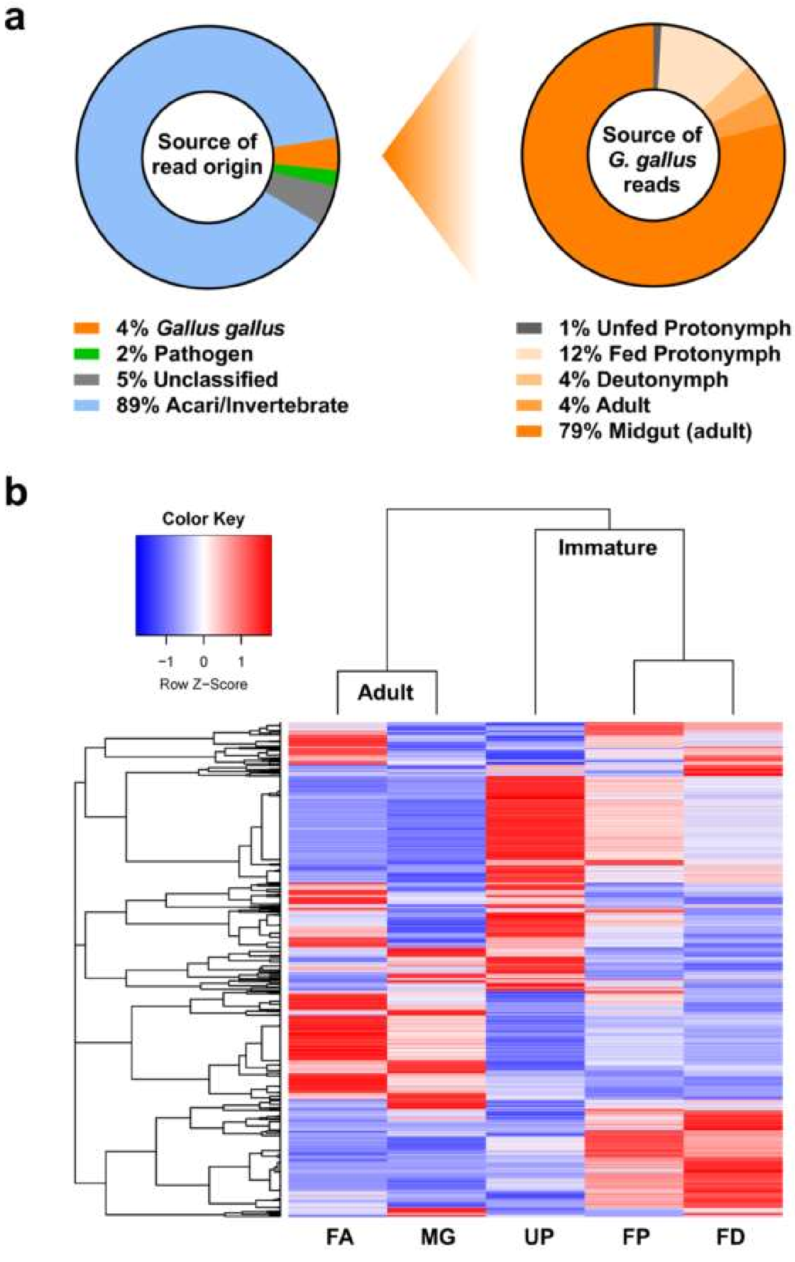

Supplementary Figure S1. **a** Attributed origin of the reads, from all libraries, according to the matched organism (left pie chart). The library origin of the chicken (*Gallus gallus*)-derived contigs is shown in the right pie chart. **b** Heat map for 12,484 selected transcripts showing diversity of transcripts expressions among studied libraries. UP - unfed protonymph, FP - fed protonymph, FD - fed deutonymph, FA - fed adult, MG - midgut.

Supplementary Fig. S2:

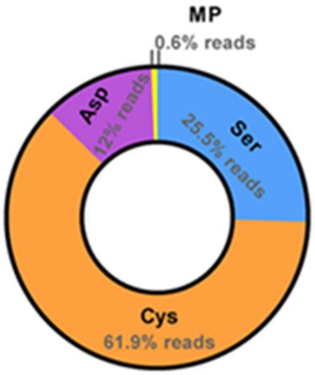

enzyme:	CathB	CathC	CathL	Legumain	CathD
query:	XP_015782231.1	ABV29335.2	ABO26562.1	AQX35828.1	JAB70978.1
Lp	XP_013771910.1	XP_013792052.1	XP_022236235.1	XP_013783249.1	XP_013790156.1
Pt	XP_042911940.1	XP_035206653.1	XP_042899502.1	XP_042908679.1	XP_015911491.1
Tu	XP_015782231.1	Eval: 4e-19	XP_015790859.1	XP_015788787.1	XP_015792854.1
Ss	KAF7494869.1	Eval: 2e-20	KAF7492022.1	Eval: 7e-27	KAI7697149.1
Is	XP_040066841.1	XP_029843222.2	XP_029838979.1	XP_040073601.2	XP_042143440.1
Rm	QTX16298.1	XP_037273909.1	XP_037281048.1	XP_037285162.1	XP_037284756.1
Mo	Eval: 2e-13	Eval: 8e-19	XP_003743778.1	XP_003746513.1	XP_018497450.1
Vd	Eval: 6e-14	Eval: 1e-17	XP_022650879.1	Eval: 1e-29	XP_022687503.1
*Nc**	Eval: 8e-11	Eval: 8e-12	Eval: 3e-109	Eval: 1e-59	Eval: 2e-83
Dg	Eval: 8e-17	Eval: 3e-18	MBD2885923.1	MBD2885742.1	MBD2876189.1

* tBlastN wgs: BioProject: 549381

Supplementary Figure S2. Left: A pie chart of the partition of individual protease families identified in the *D. gallinae* transcriptome; Asp, Cys, Ser, MP - Aspartyl, Cysteine, Serine proteases, and Metalloproteases. **Right:** BlastP results of known cysteine and aspartic proteases against selected Chelicerates with a specific focus on Acari species. Accession IDs of homologues identified, as well as query sequences used, presented in Figure 3a are shown here. *Lp*, *Limulus polyphemus*; *Pt*, *Parasteatoda tepidariorum* (a representative of Araneae); *Tu*, *Tetranychus urticae*; *Ss*, *Sarcoptes scabiei*; *Is*, *Ixodes scapularis*; *Rm*, *Rhipicephalus microplus*; *Mo*, *Metaseiulus occidentalis*; *Vd*, *Varroa destructor*; *Nc*, *Neoseiulus cucumeris*; Dg, *Dermanyssus gallinae*.

Supplementary Fig. S3:

a

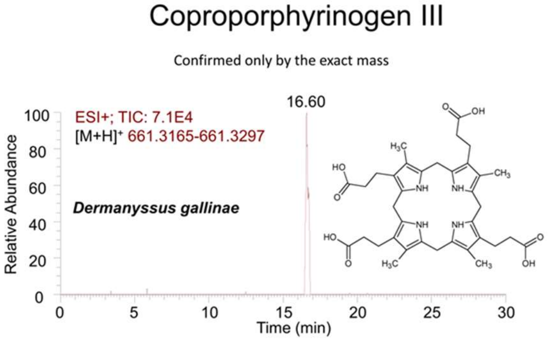

b

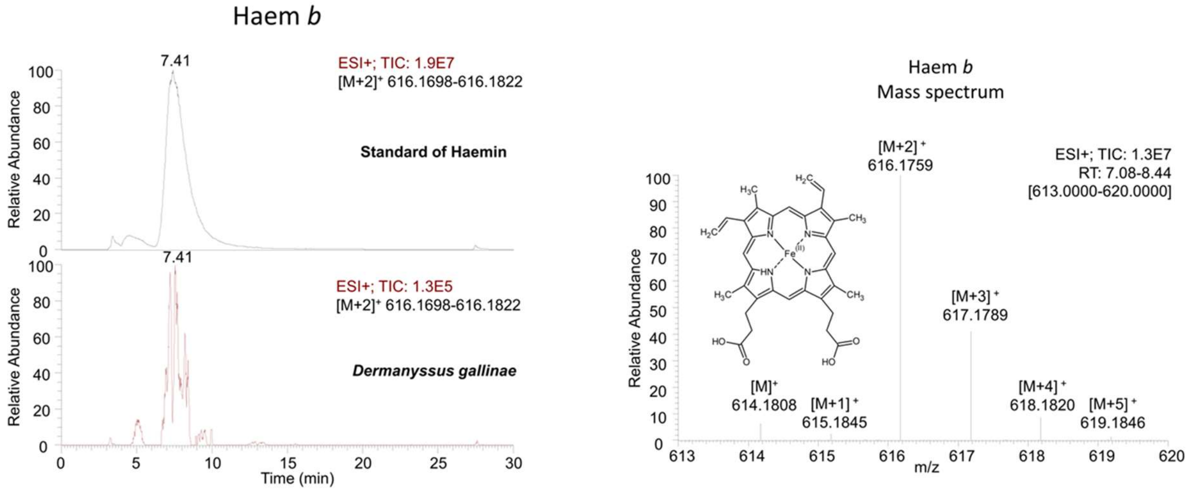

Supplementary Figure S3. a The reconstructed chromatogram for Coproporphyrinogen III $[M+H]^+$ 661.3165-661.3297 for *Dermanyssus gallinae* samples, confirmed only by the exact mass. **b** The reconstructed chromatogram for Haem *b* $[M+2]^+$ 616.1698-616.1822, first for standard (Haemin) and second for *Dermanyssus gallinae* sample and mass spectrum of Heamin for explanation of diagnostic mass $[M+2]^+$.

Supplementary Fig. S4:

Dg Ferritin 1 - intracellular

Putative iron responsive element

```
gcctcggttagccttgggtcaacagtgcttgaacgggctgatctggcagacacgttcgac

ttaacgactgaactcagcattatcatcgtgataccaccgaagagacggcttgaagctgcc

caggaagattaactgccttcactgagtagtgcaacagccttgtcgttggtgaccaaaggt

tctcaagttcctttcgtctagcgttgtcgttgttctgagccgccttcaccgccagccaac

atgtcgaccgcgtcgcgtccccgtcagaacttccacgctgattgcgaggatgcaatcaac
 M  S  T  A  S  R  P  R  Q  N  F  H  A  D  C  E  D  A  I  N
aagcaaatcaacatggagctctacgcctcctacgtctacatgtcgatgggcttttacttc
 K  Q  I  N  M  E  L  Y  A  S  Y  V  Y  M  S  M  G  F  Y  F
gatcgtgatgacgtggcgttcaagaacgtgaagaagtttttccttaaggcttctgaggag
 D  R  D  D  V  A  F  K  N  V  K  K  F  F  L  K  A  S  E  E
gagagggaacacgccatgaagctcatgtcataccagaatatgcgcggcggacgcattgtg
 E  R  E  H  A  M  K  L  M  S  Y  Q  N  M  R  G  G  R  I  V
ctgcagcctgtggctaagccggaacgcgatgagtgggattctattcagcaggcttttgag
 L  Q  P  V  A  K  P  E  R  D  E  W  D  S  I  Q  Q  A  F  E
tcggccatcgaacttgagaaaaaggtcaacgagagtctgctgaatatccacaagatcgcc
 S  A  I  E  L  E  K  K  V  N  E  S  L  L  N  I  H  K  I  A
agtgatcatgcggacgcccagttctgcgacttcctcgagacacactaccttgaggaacag
 S  D  H  A  D  A  Q  F  C  D  F  L  E  T  H  Y  L  E  E  Q
gtgaaatcgatcaagcagctgtctgatcacatcacgaacattatacgcgtcggcagtggc
 V  K  S  I  K  Q  L  S  D  H  I  T  N  I  I  R  V  G  S  G
ctcggcgagttcatcttcaacaaggagctcggcgacgactaa
 L  G  E  F  I  F  N  K  E  L  G  D  D  -
```

Dg Ferritin 2 - secretory

```
atgaaacgtctcgtctgcatcctcgtaccgctgctcttcgcagctgtgctcgctcgccaa
 M  K  R  L  V  C  I  L  V  P  L  L  F  A  A  V  L  A  R  Q
gattcctctgtgtggttcacagggcttagcggggctggcaaaaaggcgaaccaggctgat
 D  S  S  V  W  F  T  G  L  S  G  A  G  K  K  A  N  Q  A  D
gctgtgagactcaaacgcgctcctagggctgtagataatgccaccccatcgggcaatacg
 A  V  R  L  K  R  A  P  R  A  V  D  N  A  T  P  S  G  N  T
aacaaatacgtgctcgaggacagctgtctgggggcttgcgagcccagctaaacctcgag
 N  K  Y  V  L  E  D  S  C  L  G  G  L  R  A  Q  L  N  L  E
atgcacgcctcgctcctctaccaacagatggccgcacactttgactccaaccaggttgcc
 M  H  A  S  L  L  Y  Q  Q  M  A  A  H  F  D  S  N  Q  V  A
cacaagggcttcgctaagttcttcgagaagagttcggacgaggagcgtgagcacgcgaag
 H  K  G  F  A  K  F  F  E  K  S  S  D  E  E  R  E  H  A  K
aaaatcaccaactacattaacgcacgtggcggcaccatcggccatctcaacatacggatg
 K  I  T  N  Y  I  N  A  R  G  G  T  I  G  H  L  N  I  R  M
ccgagctccaactcatgggatacggcaaaagaggcccttcaagccgccctagtcctcgag
 P  S  S  N  S  W  D  T  A  K  E  A  L  Q  A  A  L  V  L  E
catcacgtcaacaacgagttgcaccttctccacagaacggccgacgaggactgtcgtgat
 H  H  V  N  N  E  L  H  L  L  H  R  T  A  D  E  D  C  R  D
ccccagttgcaggacttcttagagagcaacttcctcagcgagcaggtggaatccatcgct
 P  Q  L  Q  D  F  L  E  S  N  F  L  S  E  Q  V  E  S  I  A
cagattgagcgtttgataacgaacctcaacaagttcggtgatgtgcatctcggagaatac
 Q  I  E  R  L  I  T  N  L  N  K  F  G  D  V  H  L  G  E  Y
tttgtcaacaaggaccatctctag
 F  V  N  K  D  H  L  -
```

Supplementary Figure S4. Intracellular and secretory ferritins from *D. gallinae* – cDNA and deduced amino-acid sequences. Putative Iron-Responsive-Element in *D. gallinae* 5' UTR of Ferritin 1 transcript: Green, the "head" part of the stem-loop structure; violet, complementary bases forming the stem; Signal peptide of secretory ferritin 2 is highlighted in yellow.

Supplementary Fig. S5:

a

	libraries -------- transcripts	Unfed Protonymph	Fed Protonymph	Fed Deutonymph	Fed Adult	Midgut (adult)	overview	Protein Link	Protein ID
Complex	*mTOR*	19,5	17,1	14,6	14,8	9,7		Dg-569977_FR6_51-2668	MBD2884038.1
	Raptor	29,2	18,1	12,4	14,7	8,3		Dg-679943_FR5_1-1337	MBD2889451.1
	Rictor	15,8	16,6	13,3	9,6	8,7		IrSigP-380157_FR2_397-1973	MBD2888345.1
Interactors	*raga*	48,5	45,6	38,4	74,1	91,1		Dg-407637_FR2_180-488	MBD2884893.1
	ragc	60,2	58,7	70,6	94,8	123,5		IrSigP-551279_FR5_1238-1716	MBD2875944.1
Substrates	*S6K*	74,3	77,4	81,9	78,3	80,1		Dg-361189_FR3_761-1234	MBD2877919.1
	4E-BP	950,0	261,7	150,7	283,9	169,6		Dg-409360_FR3_337-498	MBD2883246.1
	AKT	92,6	68,2	61,7	66,3	44,6		Dg-369246_FR2_19-555	MBD2884288.1
IIS	*InR*	30,2	24,3	22,0	17,9	9,2		Dg-656129_FR5_567-2204	MBD2883038.1
	ilp4	38,4	24,3	25,9	7,9	2,6		IrSigP-1069941	MBD2883068.1
	ilp7	108,4	50,3	24,3	4,5	1,1		IrSigP-787143	MBD2882243.1
	Insulinase	92,0	110,5	86,3	127,4	137,4		IrSigP-393788	MBD2890617.1

b

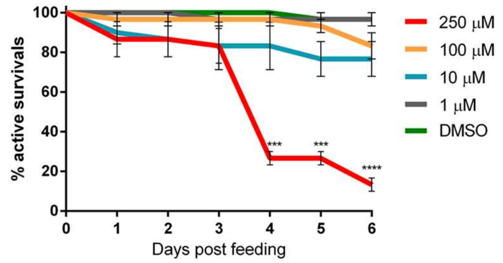

Supplementary Figure S5. Overview of transcripts encoding proteins implicated in nutrient status signaling pathways. IIS, insulin/IGF signaling; InR, insulin receptor; Ilp, insulin-like petide. **a** Mapping of nutrient sensing and storage, and TOR-mediated signaling pathway in *D. gallinae* developmental stages, using *Caenorhabditis elegans* homologues, FPKMs and accession numbers are shown. **b** Survival plot of mites exposed to Torin2 inhibitor, through an *ex vivo* membrane feeding system. Each value is derived from 10 engorged mites in the feeding chamber; Mean and SEM from three independent chambers are shown from two independent experiments. Statistical t-test indicate *** p = 0.0001 and **** p < 0.0001.

Supplementary Fig. S6:

```
              Signal peptide
MBD2887064    ---------- ---------- ---------- ---------- ---------- ---------- ---------- 70
MBD2876840    MSVNIQNALS SAMYILLGTI LLGSATVLAS ALSPNHHKQQ QMQEFAGVSS PSLYQQPLPL PQAIRGKPKS
MBD2886266    MSVNIQNALS SAMYILLGTI LLGSATVLAS ALSPNHHKQQ QMQEFAGVSS PSLYQQPLPL PQAIRGKPKS
MBD2879406    MSVNIQNALS SAMYILLGTI LLGSATVLAS ALSPNHHKQQ QMQEFAGVSS PSLYQQPLPL PQAIRGKPKS
NP_507090.    MATWIVGKLI IASLILGIQA QQARTKSQDI FEDDNDNGTT TLESLARLTS P--------- ----------
NP_491470.    ---------- ---------- -MTTPSSFSI L--------- ----LLLLIM P--------- ----------

MBD2887064    --MTSDKEGK ANFRAIEKRI LDSIIGVGRY DSRIRPMGIN N-TDGPALVR VNIYIRSIGK IDDVTMEYTV 140
MBD2876840    PYMTSDKEGK ANFRAIEKRI LDSIIGVGRY DSRIRPMGIN N-TDGPALVR VNIYIRSIGK IDDVTMEYTV
MBD2886266    PYMTSDKEGK ANFRAIEKRI LDSIIGVGRY DSRIRPMGIN N-TDGPALVR VNIYIRSIGK IDDVTMEYTV
MBD2879406    PYMTSDKEGK ANFRAIEKRI LDSIIGVGRY DSRIRPMGIN N-TDGPALVR VNIYIRSIGK IDDVTMEYTV
NP_507090.    --IHIPIEQP Q---TSDSKI LAHLFTSG-Y DFRVRPPTDN G---GPVVVS VNMLLRTISK IDVVNMEYSA
NP_491470.    --VVTNGEYS M---QSEQEI LNALLKN--Y DMRVRPPPAN SSTEGAVNVR VNIMIRMLSK IDVVNMEYSI

MBD2887064    QLTFRQQWRD ERLQYDDLG- --GQIRYLTL TDQKKLWQPD LFFSNEKEGH LHQIIVPNVL LRIYPNGDVL 210
MBD2876840    QLTFRQQWRD ERLQYDDLG- --GQIRYLTL TDQKKLWQPD LFFSNEKEGH LHQIIVPNVL LRIYPNGDVL
MBD2886266    QLTFRQQWRD ERLQYDDLG- --GQIRYLTL TDQKKLWQPD LFFSNEKEGH LHQIIVPNVL LRIYPNGDVL
MBD2879406    QLTFRQQWRD ERLQYDDLG- --GQIRYLTL TDQKKLWQPD LFFSNEKEGH LHQIIVPNVL LRIYPNGDVL
NP_507090.    QLTLRESWID KRLSYGVKGD GQPD--FVIL TVGHQIWMPD TFFPNEKQAY KHTIDKPNVL IRIHNDGTVL
NP_491470.    QLTFREQWID PRLAYENLGF YNPPAFLTVP HVKKSLWIPD TFFPTEKAAH RHLIDMENMF LRIYPDGKIL

                         Cys loop
MBD2887064    FSIRISLVLS CPMNLKFYPL DKQICSILMA SYGYTTEDLV FLWKEGDPVQ VTKNLH--LP RFTLER-FNT 280
MBD2876840    FSIRISLVLS CPMNLKFYPL DKQICSILMA SYGYTTEDLV FLWKEGDPVQ VTKNLH--LP RFTLER-FNT
MBD2886266    FSIRISLVLS CPMNLKFYPL DKQICSILMA SYGYTTEDLV FLWKEGDPVQ VTKNLH--LP RFTLER-FNT
MBD2879406    FSIRISLVLS CPMNLKFYPL DKQICSILMA SYGYTTEDLV FLWKEGDPVQ VTKNLH--LP RFTLER-FNT
NP_507090.    YSVRISLVLS CPMYLQYYPM DVQQCSIDLA SYAYTTKDIE YLWKEHSPLQ LKVGLSSSLP SFQLTN-TST
NP_491470.    YSSRISLTSS CPMRLQLYPL DYQSCNFDLV SYAHTMNDIM YEWDPSTPVQ LKPGVGSDLP NFILKNYTTN

                                          out       TM1          in           TM2
MBD2887064    DYCTSRTNTG EYSCLKVDLV FKREFSYYLI QIYIPCCMLV VVSWVSFWLD PTSIPARVSL GVTTLLTMAT 350
MBD2876840    DYCTSRTNTG EYSCLKVDLV FKREFSYYLI QIYIPCCMLV VVSWVSFWLD PTSIPARVSL GVTTLLTMAT
MBD2886266    DYCTSRTNTG EYSCLKVDLV FKREFSYYLI QIYIPCCMLV VVSWVSFWLD PTSIPARVSL GVTTLLTMAT
MBD2879406    DYCTSRTNTG EYSCLKVDLV FKREFSYYLI QIYIPCCMLV VVSWVSFWLD PTSIPARVSL GVTTLLTMAT
NP_507090.    TYCTSVTNTG IYSCLRTTIQ LKREFSFYLL QLYIPSCMLV IVSWVSFWFD RTAIPARVTL GVTTLLTMTA
NP_491470.    ADCTSHTNTG SYGCLRMQLL FKRQFSYYLV QLYAPTTMIV IVSWVSFWID LHSTAGRVAL GVTTLLTMTT

                                     TM3
MBD2887064    QISGINASLP PVSYTKAIDV WTGVCLTFVF GALLEFALVN YASRSDQRRQ NIHKQSVPQA SQQRQKWLLP 420
MBD2876840    QISGINASLP PVSYTKAIDV WTGVCLTFVF GALLEFALVN YASRSDQRRQ NIHKQ----A SQQRQKWLLP
MBD2886266    QISGINASLP PVSYTKAIDV WTGVCLTFVF GALLEFALVN YASRSDQRRQ NIHKQSVPQA SQQRQKWLLP
MBD2879406    QISGINASLP PVSYTKAIDV WTGVCLTFVF GALLEFALVN YASRSDQRRQ NIHKQ----- ----------
NP_507090.    QSAGINSQLP PVSYIKAIDV WIGACMTFIF CALLEFALVN HIANKQGVER KARTE----- ----------
NP_491470.    MQSAINAKLP PVSYVKVVDV WLGACQTFVF GALLEYAFVS YQDSVRQNDR SREKA----- ----------

MBD2887064    ASIAAAGLEN NQSAIDHCDE AFAMPFQGSA AERGGMARNA LPNDADILVP RQRTHTAPPQ EIRQCEVHMK 490
MBD2876840    ASIAAAGLEN NQSAIDHCDE AFAMPFQGSA AERGGMARNA LPNDADILVP RQRTHTAPPQ EIRQCEVHMK
MBD2886266    ASIAAAGLEN NQSAIDHCDE AFAMPFQGSA AERGGMARNA LPNDADILVP RQRTHTAPPQ EIRQCEVSST
MBD2879406    ---------- ---------- ---------- ---------- ---------- ---------- ------VCIT
NP_507090.    ---------- ---------- ---------- ---------- -REKAEIPLL QNLHNDVPTK VFNQEEKVRT
NP_491470.    ---------- ---------- ---------- ---------- -ARKAQR--R REKLEMVDAE VYQPPCTCHT

                                            in          TM4         out
MBD2887064    GPSSTSSNLC RSWLSRFPTR S----KRIDV ISRIFFPLMF ALFNLVYWTT YLFREDAD-- ----- 555
MBD2876840    GPSSTSSNLC RSWLSRFPTR S----KRIDV ISRIFFPLMF ALFNLVYWTT YLFREDAD-- -----
MBD2886266    SPRQHTALFC CLIYYNYFTG N----SDLIV NSVLKVNIIF RMS----WQL AIIGT----- -----
MBD2879406    CSYTNSLLVV LVFLYIILQS Y----STINL N--------- ---------- ---------- -----
NP_507090.    VPLNRRQMNS FLNLLETKTE WNDISKRVDL ISRALFPVLF FVFNILYWS- ------RFGQ QNVLF
NP_491470.    FEA-RETFRD KVRRYFTKPD Y--LPAKIDF YARFVVPLAF LAFNVIYWVS CLIMSANAST PESLV
```

Supplementary Figure S6. Amino acid alignment of identified sequences of *D. gallinae* glutamate-gated chloride channels (in bold). Alpha and beta subunits of *C. elegans* homologues (NP_507090 and NP_491470, respectively) served as templates for identifications of secondary structures. Residues involved in interactions with ivermectin are highlighted in green.

Supplementary Fig. S7:

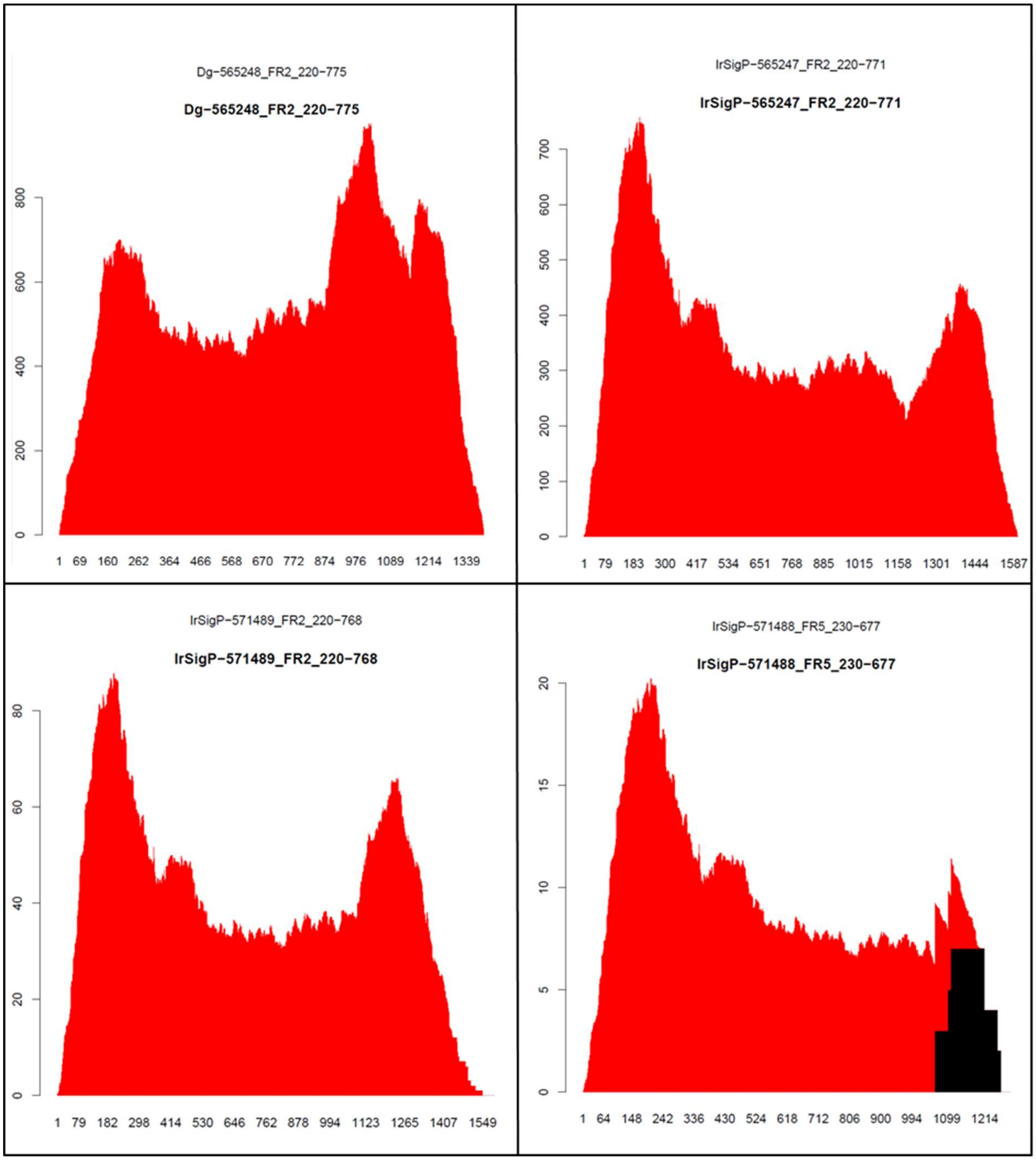

Supplementary Figure S7. Visualisation of read coverage of glutamate-gated chloride channels encoding transcripts, assessed through RNA-Seq by Expectation-Maximization (RSEM) software package. Red indicates aligned read assignment over multiple transcripts, while black indicates uniquely assigned reads.

Supplementary Fig. S8:

Type	Thiol	Protein-ID[a]	RNAseq-ID	Unfed Protonymph FPKM	Fed Protonymph FPKM	Deutonymph FPKM	Adult FPKM	Midgut (adult) FPKM
**DgA2M(sv1)**	Yes	MBD2876438.1	Dg-350935_FR6_221-1770	193,66	78,56	60,35	59,62	76,14
**DgA2M(sv2)**	Yes	MBD2883834.1	IrSigP-350924_FR6_98-1629	23,34	120,29	149,36	47,93	20,13
**DgA2M(sv3)**	Yes	MBD2876440.1	IrSigP-350933_FR6_221-1778	15,71	11,66	12,86	0,33	0,86
**DgA2M(sv4)**	Yes	MBD2879613.1	IrSigP-1160473_FR1_175-1366	8,87	20,43	20,51	6,63	7,4
**DgA2M(sv5)**	Yes	MBD2878485.1	Dg-350932_FR3_95-1489	0,1	11,37	8,72	9,18	9,41
**DgC3**	Yes	MBD2883842.1	IrSigP-433789_FR5_615-2320	39,31	21,25	23,05	27,66	28,98
**DgTEP**	Yes	MBD2879163.1	IrSigP-580656_FR2_299-1580	33,55	77,78	83,56	26,9	8,46
**DgMCR-1**	No	MBD2885045.1	Dg-370713_FR6_393-2047	12,62	12,64	15,08	1,62	0,17
**DgMCR-2**	Yes	MBD2885046.1	IrSigP-392592_FR1_123-1721	16,12	36,15	28,07	0,98	0,06
**DgMCR-3**	No	MBD2882826.1	Dg-594457_FR6_1037-2807	7,2	4,1	5,21	0,26	0,02

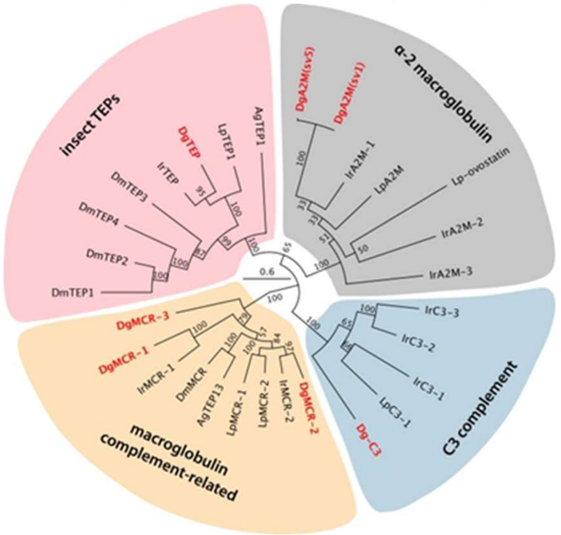

Alternative splicing within the 'bait regions' of *D. gallinae* α₂-macroglobulins:

```
DgA2M(sv5)   1 PCRSTRDGSHTSVPELGVTLEELA------------GPM------------------ATGTTYAAAPSASRIGNDFGGATATASQSVVEVRDYFPELW
DgA2M(sv4)   1 PCRSTRDGSHTSVPELAPAREAAMVEFDS-------EPAADGDDDTAAADRDRLVRPQTTFSTTAAPSASRIGNDFGGATATASQSVVEVRDYFPELW
DgA2M(sv3)   1 PCRSTRDGSHTSVPELVSKRNGFVPTRPLAVSGHIAGPSLAAHRPAAAARPPPIPR---PPPAAAAPSASRIGNDFGGATATASQSVVEVRDYFPELW
DgA2M(sv1)   1 PCRSTRDGSHTSVPERVSLKMVAFST----------------------------SS---GERRFAAPSASRIGNDFGGATATASQSVVEVRDYFPELW
DgA2M(sv2)   1 PCRSTRDGSHTSVPEP-------------------------------------------------APSASRIGNDFGGATATASQSVVEVRDYFPELW
```

Supplementary Figure S8. Identified transcripts encoding *D. gallinae* members of thiol-ester containing protein (TEP) family and phylogenetic tree with selected well-annotated TEPs from other invertebrates. The tree was reconstructed using the maximum likelihood method based on the alignment of full amino acid sequences (~1500 residues). Ag – malaria mosquito *Anopheles* gambiae; Dg (in red) – red poultry mite *Dermanyssus gallinae;* Dm – the fruit fly *Drosophila melanogaster*; Ir – the hard tick *Ixodes ricinus;* Lp – the horseshoe crab *Limulus polyphemus.* Numbers at the branches represent bootstrap support values calculated from 1,000 replicates. **GenBank Accession Nos. to the TEP sequences of other invertebrates shown in the phylogenetic tree:** AgTEP1 (AVK92986.1); AgTEP13 (XP_317044.4); DmTEP1 (CAB87807.1); DmTEP2 (CAB87808.1); DmTEP3 (CAB87809.1); DmTEP4 (CAB87810.1); DmMCR (AAF52601.1); IrA2M-1 (QOJ54010.1); IrA2M-2 (QOJ54011.1); IrA2M-3 (QOJ54012.1); IrC3-1 (QOJ54014.1); IrC3-2 (QOJ54015.1); IrC3-3 (QOJ54016.1); IrMCR-1 (QOJ54017.1); IrMCR-2 (QOJ54018.1); IrTEP (QOJ54013.1); LpA2M (BAA19844.1); LpC3-1 (XP_013775397.1); LpMCR-1 (XP_022254489.1); LpMCR-2 (XP_013776899.1); Lp-ovostatin (XP_022249030.1); LpTEP1 (XP_013786421.1).Expression values (FPKM) are shown in the Table below, [a] blue Accession Nos. are used in the phylogenetic tree.

Supplementary Fig. S9:

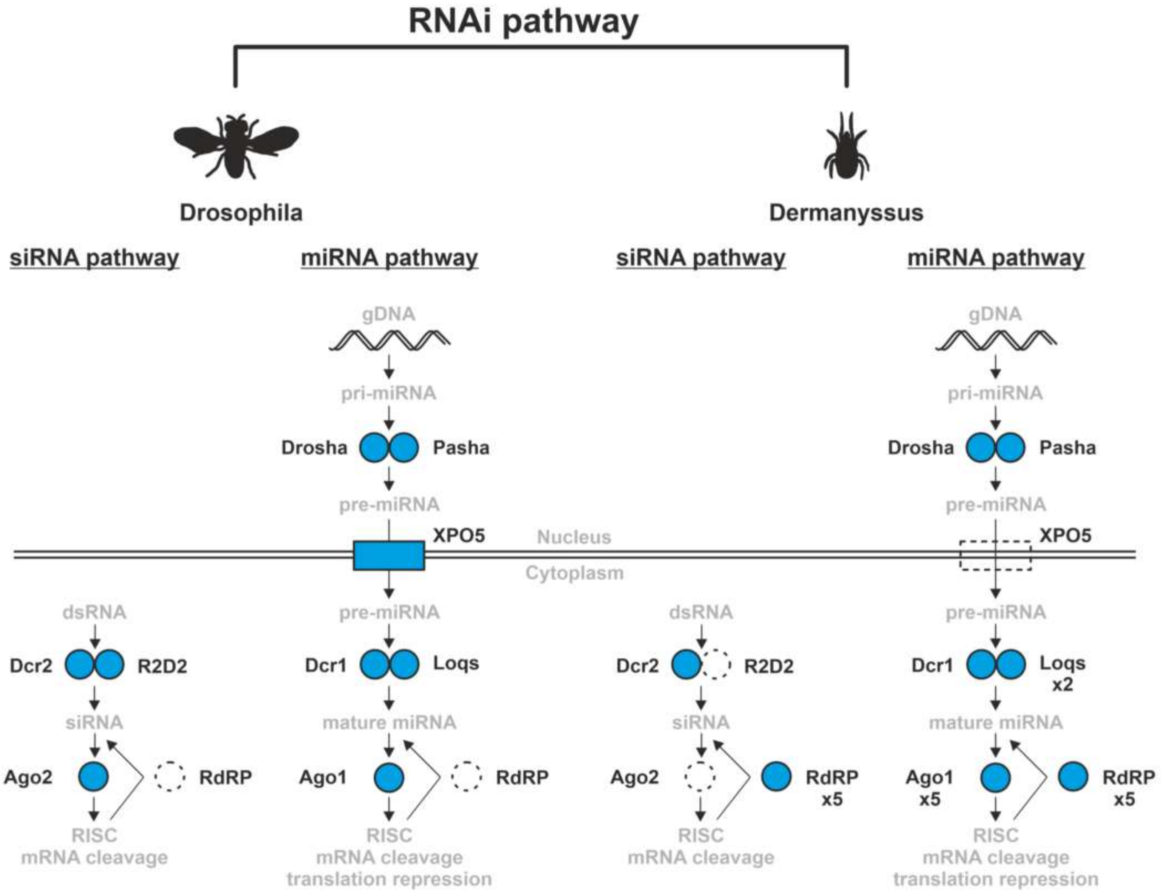

Supplementary Figure S9. Identified transcripts encoding components of RNAi pathway in the *D. gallinae* transcriptomes. Protein sequences of *Drosophila* or *Tribolium* (Tube) RNAi pathway were used to search in our *D. gallinae* translated protein database by using program Bioedit (Local BLAST, E value 0.1, Matrix BLOSUM62). Conservation of the domains was checked by CD-search (NCBI). Only hits with similar domain structures and an E-value < $10e^{-3}$ were considered as putative homologues. Accession numbers are available as **Supplementary Table S9**.

Supplementary Table S1: Information on read counts and quality scores achieved by Illumina sequencing.

Sample	Raw Reads	Effective Rate (%)	GC content (%)	Error Rate (%)	Q20 (%)	Q30 (%)
unfed protonymphs	57338502	97.58	49.19	0.03	97.89	94.09
fed protonymphs	58861591	97.54	49.69	0.03	97.79	93.90
deutonymphs	58727555	97.99	49.87	0.03	97.85	94.00
adults	54699347	97.73	50.75	0.02	98.14	94.77
midguts from adults	61346035	98.09	49.51	0.03	97.90	94.33

Supplementary Table S2: Summary of reads origin.

	Sum of reads	% total reads	Contigs	% total contigs
REFSEQ-INVERTEBRATE	126,948,051	41.52	19,590	23.02
ACARI	153,704,406	50.27	11,739	13.79
GALLUS	3,301,469	1.08	11,258	13.23
TICK-PATHOGENS	6,432,162	2.10	4,251	4.99
UK	15,389,923	5.03	38,279	44.97
Total	**305,776,011**	**100**	**85,117**	**100**

Supplementary Table S3: Accession IDs to differentially expressed transcripts in blood-fed protonymphs over transcriptomes of unfed protonymphs, shown in Figure 2c. Oligoes used in RT-qPCR in Figure 2c' are shown in the last column.

DET No	Annotation	Transcript ID (hyper-linked Excel file)	Protein ID (NCBI)	RT-qPCR primers
2	Carboxypeptidase	IrSigP-456705_FR5_11-515	MBD2876455	F: GAGAATCGCCAGCAGAACCT<br>R: CGTATGGTACTGGGTCAGGC
3	717131_FR2_65-201	Dg-717131_FR2_65-201	MBD2886605	F: AAACCTTTGGCAGCGTACTC<br>R: CGCTCCCTCGGTAGAAATCT
4	465142_FR6_1943-2222	IrSigP-465142_FR6_1943-2222	MBD2879726	F: GACGAGAAGGGTTTGTCCTC<br>R: TGTCGAGGTAGATGTGCTGG
5	Stubble	IrSigP-702379_FR6_1752-2725	MBD2890242	F: TGATCGGCATCATCTCGTGG<br>R: CCTCGTGATGCGGGTGTAAA
6	Cathepsin L5	Dg-417098_FR1_205-573	MBD2876666	F: GTGTTCGATCCGTGGTTCTG<br>R: AGTTCTTCACGAGCCAGGAG
7	Cuticle protein 7	IrSigP-633645_FR4_104-234	MBD2890009	F: CGATTACCGGCCGAGTCTAC<br>R: GGCGCTATGTACGAGGTGAC
8	464593_FR5_41-172	IrSigP-464593_FR5_41-172	MBD2874827	F: TGTTTGCTCACATGGTACGC<br>R: GATCTTTCCGGCAGCTTAGC
9	Chitinase-like	Dg-426972_FR5_591-1070	MBD2887237	F: ACAGAAAAGACCTGGTGGCC<br>R: CACCAGGTTTTCTCCGTCGA
10	Neyo-like	Dg-429223_FR6_180-923	MBD2886979	F: TGGCGTTGACAATTCCGAGA<br>R: GTACAGAGGCCCCATGATGG
11	Astacin-like metalloprotease	Dg-768351_FR2_357-560	MBD2887970	F: ACGGTGGTTCACGAGTTTGG<br>R: GCTTGATGAATTGGTCGCGG
12	703171_FR2_257-506	IrSigP-703171_FR2_257-506	MBD2883573	F: GAAGCCGCTGTGAAGCTAGA<br>R: GCACTGCGCATAGATCTGGA
13	Mucin	Dg-385271_FR5_1-555	MBD2881848	F: AGTACCAGGCTCCTCAACCT<br>R: TATAGGCAGGTTGACGGGGA
14	Peritrophin 1	IrSigP-436463_FR6_542-720	MBD2890592	F: GTGCTTTCAGTCGCCGTATG<br>R: CAAGTTGCTTCACCTGACGC
15	Peritrophin 2	IrSigP-436466_FR2_223-511	MBD2889708	F: GGCACCTACTTTGTTTCGCA<br>R: CACGTGATAGAATGGGCAGC
	Legumain isoenzyme 4	IrSigP-379957_FR2_1085-1545	MBD2885568	F: CCATAGCGGTGTGTGTCCTC<br>R: GTTTGGCCACCATCTTCAGC

Supplementary Table S4: Accession IDs to differentially expressed transcripts in midguts over whole bodies of adult mites, shown in Figure 2d. Oligoes used in RT-qPCR in Figure 2d' are shown in the last column.

DET No	Annotation	Transcript ID (hyper-linked Excel file)	Protein ID (NCBI)	RT-qPCR primers
1	Trypsin-like serine protease	IrSigP-562590_FR6_227-579	MBD2888300	F: CGGACGGACTTCTTTGACGA<br>R: GAACGCTACTTCCGCTCGTA
2	Caspase-1-like isoform X2	Dg-598524_FR2_222-566	MBD2885764	F: AGGTATTCTGGCCACTGACC<br>R: TAGCCGGCCACAGTTGAATA
3	Amino acid transporter	Dg-370073_FR3_533-1060	MBD2877231	F: ACACCAATGCCAGCACTCAT<br>R: TTAGTTGGCCAATGTCCGCA
4	Apolipophorins-like	Dg-404344_FR1_98-2680	MBD2886271	F: TCTCGACATCGTTGATCGGC<br>R: CGAGTCGCTCCGTGATGATT
5	P2X purinoceptor 4	Dg-950939_FR2_274-714	MBD2886414	F: GAAGTGGAGTACAGACGGGC<br>R: CCAGCCTCGGATTTCGCATA
6	S-adenosylmethionine synthetase	Dg-620064_FR5_192-681	MBD2883952	F: GTAGTGGTGAGAGCGGTCAT<br>R: ATTGGGCCTCCTTCGATGAA
7	Ferritin 2	IrSigP-384458_FR2_148-399	MBD2883635	F: TCCTCGAGCATCACGTCAAC<br>R: GGATCACGACAGTCCTCGTC
8	Sulfotransferase family cytosolic 2B	Dg-807076_FR4_126-470	MBD2875543	F: AACGGATCAACGAGTGGAGG<br>R: GTCGTACTTCACCGAGCAGT
9	Ferrochelatase	Dg-649831_FR2_303-703	MBD2876119	F: GAGCTGGCCAAAGAGCTGAA<br>R: AGACCCCGAATGAAGTGCTC
10	Acid sphingomyelinase	IrSigP-602409_FR5_87-754	MBD2884868	F: ATCGGGGTTAACTTCGTCGC<br>R: CGTCGATACTGTAGACGCGG
11	Cathepsin D1	IrSigP-519820_FR3_359-840	MBD2876745	F: TTCAGTACGGCTCAGGATCC<br>R: ACTCCTTTGTGATCTCGCCA
12	Solute carrier family 35 member F6-like	IrSigP-662643_FR6_1690-2093	MBD2876932	F: ATATCGGGTTCGTGCCCTTC<br>R: CTTCGCGTGTCCTCCTCTTC
13	GABA receptor	Dg-565248_FR2_220-775	MBD2887064	F: CCCAACGATGCCGACATTCT<br>R: TGAACCTCACATTGCCGGAT
14	Cathepsin L3	IrSigP-413246_FR4_193-533	MBD2885740	F: GTCAAGGATCAGCAGCAGTG<br>R: TCGACAAGGTTCTGTTCGGA
15	Hemolymph lipoprotein	Dg-350407_FR6_167-1642	MBD2878204	F: GTTGCAGATGCTTGCTCGTG<br>R: TTTCGCCAGTTGTCAGCTCA

Supplementary Table S5: Accession IDs to transcripts encoding cysteine and aspartic proteases shown in Figure 3b.

Protease	isoenzyme type	Transcript ID (hyper-linked Excel file)	Protein ID (NCBI)
Serine Proteases	1. Chymotrypsin	**Dg-651195**, IrSigP-651191, Dg-651193, Dg-404258, IrSigP-651189	MBD2881020
	2. Anionic trypsin	Dg-404994	MBD2877379
	3. Chymotrypsin-like elastase	IrSigP-607270	MBD2881713
	4. Chymotrypsin-like elastase	**IrSigP-76325**, IrSigP-355347	MBD2878320
	5. Stubble-like serine protease,trypsin	IrSigP-387896	MBD2880678
	6. Stubble-like serine protease, trypsin	Dg-615055	MBD2879484
	7. Chymotrypsin	**IrSigP-417374**, Dg-417373	MBD2877373
	8. Chymotrypsin	IrSigP-622345	MBD2875824
	9. Chymotrypsin	IrSigP-997083	MBD2875973
	10. Chymotrypsin	**Dg-602081**, Dg-602079	MBD2879331
	11. Prolyl oligopeptidase	**Dg-1021459**, IrSigP-1081927	MBD2887264
	12. Serine protease K12H4.7	**IrSigP-351248**, IrSigP-351247	MBD2884270
Cysteine Proteases	1. Cathepsin L1	**Dg-632847**, IrSigP-632845	MBD2879342
	2. Cathepsin L2	**IrSigP-354374**, IrSigP-354370	MBD2878524
	3. Cathepsin L3	**IrSigP-413246**, IrSigP-10997, IrSigP-413245,	MBD2885740
	4. Cathepsin L4	**IrSigP-349742**, IrSigP-349741	MBD2885566
	5. Cathepsin L5	**Dg-417098**, Dg-417097,	MBD2876666
	6. Cathepsin L6	IrSigP-1037340	MBD2884871
	7. Legumain 1	**IrSigP-439085**, Dg-439086	MBD2885742
	8. Legumain 2	Dg-445098	MBD2886063
	9. Legumain 3	IrSigP-535196	MBD2885743
	10. Legumain 4	IrSigP-379957	MBD2885568
Aspartyl Proteases	1. Cathepsin D1	**Dg-519818**,IrSigP-476369, IrSigP-519820, IrSigP-519817	MBD2876748
	2. Cathepsin D2	**Dg-370446**, IrSigP-808266	MBD2879957
	3. Cathepsin D3	**IrSigP-16089,** Dg-1042377, Dg-1042375	MBD2876189

Supplementary Table S6: Overview of transcripts encoding proteins involved in haem and iron biology.

**Reference genome**
Aedes aegypti
Strain: Liverpool AGWG
Assembly: AaegL5
BioProject Query: 597301
tBlastN

Query protein + ID´s	transcript ID	protein ID	% coverage	E value
**ALAS**: XP_001658653.1	Dg-608857_FR3_129-736	**MBD2882839.1**	91	0.0
**PBGS**: XP_001661237.1	Dg-568812_FR5_621-1005	**MBD2885691.1**	98	2e-133
**HMBS**: XP_001654431.1	Dg-541660_FR4_65-424	**MBD2879167.1**	54	5e-82
**UROS**: XP_001660743.2	Dg-987039_FR1_193-481	**MBD2880820.1**	94	1e-33
**UROD**: XP_001657958.2	Dg-478956_FR4_182-541	**MBD2884748.1**	83	6e-126
**CPOX**: XP_001663577.1	Dg-945296_FR1_180-609	**MBD2883466.1**	87	2e-151
**PPOX**: XP_021693143.1	IrSigP-605064_FR5_94-622	**MBD2885503.1**	99	1e-93
**FECH**: XP_001650846.1	**Dg-649831_FR2_303-703** / Dg-381015_FR5_530-916	**MBD2876119.1**	94	2e-149
**HO:**				
XP_001658955.1 (aedes)	NI (not identified)			0.0001
GETP01214966.1 (varroa)	NI			0.0001
Fer1 (Ir-108507)	Dg-367272_FR4_390-576	**MBD2884432.1**	99	1,00E-80
Fer2 (Ir-108441)	IrSigP-384454_FR3_720-940/IrSigP-384458_FR2_148-399/IrSigP-384452_FR6_307-551/IrSigP-384456_FR6_68-245/Dg-384450_FR2_2079-2251	**MBD2876852.1**	82	6,00E-62
IRP (c-Aconitase, Ir-109153)	Dg-351924_FR3_573-1476	**MBD2876478.1**	100	0
Insect transferrins				
Tf1 (XP_001647719)	NI		37	1,00E-14
Tf2 (XP_021699709)	IrSigP-523377_FR5_87-930	**MBD2877432.1**	93	0,00E+00
Tf3 (EAT34844)	NI		45	2,00E-18
Tf4 (XP_001661801)	NI		51	5,00E-15

Supplementary Table S7: Accession IDs to transcripts encoding adult-specific transcripts shown in Figure 5b. Oligoes used in RT-qPCR in Figure 5b' are shown in the last column.

DET No	Annotation	Transcript ID (hyper-linked Excel file)	Protein ID (NCBI)	RT-qPCR primers
1	Vitellogenin 1	IrSigP-350331_FR2_207-2055	MBD2876257	F: GTCTCAAGCAACGCGAACTG<br>R: AACGGAGATGCGTACTTCCC
2	Vitellogenin 2	Dg-9795_FR3_189-2089	MBD2876256	F: CCCGTCAACGAGGAACTTCA<br>R: GAGTCCACATGCTGTTGGGA
3	Vitellogenin receptor	Dg-353835_FR3_42-1900	MBD2877430	F: GTGGTGGAGGTGGTAGCTTC<br>R: CCTCCTTTGGTGTTCTCGCA
4	Vitellogenin-like	IrSigP-349783_FR3_1-1870	MBD2876000	F: TGCCTATCAAACGGGTCACT<br>R: CCGTTGAACATGACCACGAG
5	Arginine kinase	Dg-844682_FR1_111-471	MBD2886196	F: ACACGCGTGATGGACATCTT<br>R: CCGGTCAATGGGTGGTAGAC
6	Tubulin alpha-8 chain	Dg-579987_FR2_129-594	MBD2876362	F: CCCTACAATTCGGTGCTGAC<br>R: CGGCAGATGTTGTAGATGGC
7	Paramyosin-like isoform X2	Dg-573818_FR1_53-613	MBD2885035	F: ACTCAAGCTGAACGAGACCG<br>R: TTTTCACCCGTTCCTGCAGT
8	Elongation of very long chain fatty acids	IrSigP-1087573_FR5_204-473	MBD2890092	F: GACTTTCCCTCGACTCAGGC<br>R: GCAGAATTTGCGCTGAGGTC
9	Epoxide hydrolase 1	Dg-415549_FR2_82-620	MBD2877337	F: CTTATGGTTCATGGCTGGCC<br>R: ACGTCAAAAGCAAGACCGTC
10	Histone-lysine N-methyltransferase	Dg-404921_FR2_1-529	MBD2889185	F: AAGGCTGTGATCGTTCTGGA<br>R: TCTTTGGCCGAGTACCCTTT
11	Serine protease 27-like	Dg-423801_FR3_697-1274	MBD2884827	F: CCTGAAGCTCCATCCGAACT<br>R: ACTTTACGCGCCGATCTAGT

Supplementary Table S8: The list of taxa used for the phylogenetic analysis of arthropod vitellogenins.

Taxonomy		Vg1	Vg1-like	Vg2
**PARASITIFORM MITES**				
**Arthropoda; Chelicerata; Arachnida; Acari; Parasitiformes; Mesostigmata; Monogynaspida; Gamasina; Dermanyssoidea**				
Varroidae	*Varroa jacobsoni*	XP_022710380	XP_022700578	XP_022701897
	Varroa destructor	AFN88463	XP_022657753	AFN88464
Laelapidae	*Stratiolaelaps scimitus*	JABWDB010000015 (00009700-RA)	JABWDB010000015 (00009665-RA)	JABWDB010000015 (00009740-RA)
	Tropilaelaps mercedesae	OQR72561	OQR79705 + OQR72029	OQR67440
Macronyssidae	*Ornithonyssus sylviarum*	GIXZ01020088	GIXZ01000835	GIXZ01000591
Dermanyssidae	***Dermanyssus gallinae***	MBD2876257 (IrSigP-350331_FR2_207-2055)	MBD2876000.1 (IrSigP-349783_FR3_1-1870)	MBD2876256 (Dg-9795_FR3_189-2089)
**Arthropoda; Chelicerata; Arachnida; Acari; Parasitiformes; Mesostigmata; Monogynaspida; Gamasina; Phytoseioidea**				
Phytoseidae	*Amblyseius swirskii*	GHIT01048819 + GHIT01041025	absent	GHIT01045440
	Amblyseius eharai	QKO00543	absent	QBZ96191
	Neoseiulus cucumeris	AGQ56698	absent	
				AGQ56699
	Neoseiulus barkeri	ASB34115	absent	ASB34116
	Galendromus occidentalis	XP_003746410	absent	
				XP_018494463
	Euseius nicholsi	QKO00545	absent	QCX36526
**TICKS**				
**Arthropoda; Chelicerata; Arachnida; Acari; Parasitiformes; Ixodida; Ixodoidea**				
Ixodidae	*Rhipicephalus microplus*	XP_037287934	absent	
				XP_037287793
	Rhipicephalus sanguineus	XP_037527682	absent	
				XP_037528003
	Rhipicephalus haemaphysaloides	QEL09190	absent	
				GIJA01010490
	Hyalomma asiaticum	KAH6940719	absent	KAH6940634
	Dermacentor silvarum	XP_037555503	absent	
				XP_037555480
			absent	
	Dermacentor variabilis	AAW78557		ABW82681
	Amblyomma hebraeum	AGQ57039	absent	
				AGQ57040
			absent	GHLT01004136 + GHLT01003656
	Haemaphysalis longicornis	GHLT01005427		
			absent	
	Ixodes scapularis	MOY43367		MOY34203
			absent	
	Ixodes ricinus	GIDG01029412		GIDG01034908.1
			absent	
	Ixodes holocyclus	GIBQ01001668		GIBQ01041644
			absent	
Argasidae	*Ornithodoros moubata*	JAW06414		BAH02666
**ACARIFORM MITES**				
**Arthropoda; Chelicerata; Arachnida; Acari; Acariformes; Trombidiformes; Prostigmata**				
Eleutherengona; Raphignathae; Tetranychoidea; Tetranychidae	*Tetranychus urticae*	XP_015793828; XP_015793699		
	Tetranychus truncatus	AYV88983		
	Tetranychus evansi	AYV89292		

	Panonychus citri	AHN48900
Anystina; Parasitengona; Trombidioidea; Trombidiidae	*Dinothrombium tinctorium*	RWS06019; RWS07112
Anystina; Parasitengona; Trombiculoidea; Trombiculidae	*Leptotrombidium deliense*	RWS27858
**Arthropoda; Chelicerata; Arachnida; Acari; Acariformes; Sarcoptiformes; Astigmata**		
Psoroptidia; Analgoidea; Pyroglyphidae; Dermatophagoidinae	*Dermatophagoides pteronyssinus*	XP_027195573
	Dermatophagoides farinae	KAH7640524
Psoroptidia; Sarcoptoidea; Sarcoptidae; Sarcoptinae	*Sarcoptes scabiei*	KAF7496282
**SPIDERS**		
**Arthropoda; Chelicerata; Arachnida; Araneae**		
	Trichonephila clavata	GFQ69744, GFQ69748
	Parasteatoda tepidariorum	XP_015930209; XP_015921257
	Pardosa pseudoannulata	AXN69712; AXN69713
**SCORPIONS**		
**Arthropoda; Chelicerata; Arachnida; Scorpiones**		
	Centruroides sculpturatus	XP_023225368
**HORSESHOE CRAB**		
**Arthropoda; Chelicerata; Merostomata; Xiphosura**		
	Limulus polyphemus	XP_022258932; XP_022244466
**CRUSTACEANS**		
**Arthropoda; Mandibulata; Pancrustacea; Crustacea; Multicrustacea; Malacostraca; Eumalacostraca; Eucarida; Decapoda**		
	Penaeus vannamei	AAP76571
	Metapenaeus ensis	AAM48287, AAN40701, AAT01139
	Macrobrachium rosenbergii	BAB69831
	Pandalus hypsinotus	BAD11098
	Cherax quadricarinatus	AAG17936
**INSECTS**		
**Arthropoda; Mandibulata; Pancrustacea; Hexapoda; Insecta**		
	Solenopsis invicta	AAP47155; AAY22960; AAY22961
	Cimex lectularius	CLEC001325-RA; CLEC001323-RA
	Aedes albopictus	XP_019542945; XP_019542959
	Bombyx mori	NP_001037309
	Pediculus humanus corporis	XM_002431351; XM_002431353
	Tribolium castaneum	XP_970210; XP_971398
	Papilio xuthus	KPJ04900

Supplementary Table S9: Accession IDs to transcripts encoding Toll, IMD, and RNAi pathway components.

**Toll pathway**		***According to Palmer and Jiggins, MBE, 2015***		***Bioedit, local blast 0.1***			
Tagret	*Drosophila / Tribolium*	*D. gallinae*	e-value	Identities	**Protein ID**	Final number of genes	Unique sequences
PGRP-SA	NP_572727.1	Dg-540147_FR1_164-359	6,00E-40	76/163 (46%)	MBD2887001.1	3	2
		Dg-540146_FR1_117-298	6,00E-40	76/163 (46%)	MBD2886483.1		
		IrSigP-364299_FR6_987-1226	8,00E-27	57/193 (29%)	MBD2876088.1		
GNBP1	NP_524142.2	missing				0	0
GNBP3	NP_523986.2	missing				0	0
spz	NP_524526.1	Dg-700091_FR4_344-668	9,00E-11	34/108 (31%)	MBD2885047.1	11	9
		Dg-453081_FR2_746-1664	2,00E-05	33/116 (28%)	MBD2881466.1		
		Dg-1044839_FR6_83-641	2,00E-05	33/116 (28%)	MBD2881465.1		
		Dg-518881_FR1_385-2031	2,00E-05	37/152 (24%)	MBD2886331.1		
		Dg-382595_FR1_94-374	3,00E-05	30/111 (27%)	MBD2875246.1		
		Dg-382596_FR1_549-829	3,00E-05	30/111 (27%)	MBD2875239.1		
		IrSigP-382594_FR1_134-419	3,00E-05	30/111 (27%)	MBD2875245.1		
		IrSigP-512652_FR1_183-755	8,00E-05	35/146 (23%)	MBD2886337.1		
		IrSigP-1079353_FR4_183-755	8,00E-05	35/146 (23%)	MBD2886336.1		
		IrSigP-778055_FR6_423-704	8,00E-05	31/105 (29%)	MBD2890046.1		
		Dg-782185_FR4_112-753	8,00E-05	31/105 (29%)	MBD2882432.1		
Toll-1	NP_524518.1	Dg-576571_FR1_304-1394	9,00E-92	267/914 (29%)	MBD2880219.1	12	8
		Dg-567274_FR5_43-1875	2,00E-91	278/968 (28%)	MBD2879243.1		
		Dg-578655_FR2_164-1257	4,00E-91	266/909 (29%)	MBD2880160.1		
		IrSigP-382316_FR3_61-1433	1,00E-77	257/985 (26%)	MBD2882696.1		
		Dg-446710_FR5_49-1280	3,00E-73	247/971 (25%)	MBD2885043.1		
		IrSigP-446711_FR2_241-1533	4,00E-73	246/971 (25%)	MBD2883423.1		
		Dg-709305_FR5_596-2090	3,00E-71	222/852 (26%)	MBD2877998.1		
		Dg-32901_FR5_352-1601	7,00E-71	257/1002 (25%)	MBD2885673.1		
		Dg-446923_FR2_336-1582	2,00E-68	241/981 (24%)	MBD2875902.1		
		Dg-1068943_FR6_265-1548	1,00E-65	248/992 (25%)	MBD2885481.1		
		IrSigP-1142933_FR4_826-2108	7,00E-58	232/912 (25%)	MBD2885044.1		

		Dg-964834_FR3_197-1494	2,00E-57	243/1033 (23%)	MBD2883165.1		
Myd88	NP_610479.1	Dg-366848_FR6_409-694	7,00E-10	64/274 (23%)	MBD2883109.1	1	1
tube	XP_008198084 (*T. castaneum*)	Dg-1030207_FR3_288-932	1,00E-30	87/277 (31%)	MBD2875407.1	1	1
pele	NP_476971.1	Dg-520307_FR2_978-1673	1,00E-57	137/329 (41%)	MBD2887978.1	2	1
		Dg-520315_FR1_520-1491	3,00E-36	83/194 (42%)	MBD2889692.1		
dorsal	NP_724052.1	Dg-476498_FR5_529-1233	5,00E-72	175/459 (38%)	MBD2879760.1	3	1
		Dg-476504_FR3_183-907	5,00E-72	175/459 (38%)	MBD2875479.1		
		Dg-476503_FR2_303-1034	5,00E-72	175/459 (38%)	MBD2878093.1		
Dif	NP_523589.2	missing				0	0
cactus	NP_723960.1	Dg-359173_FR2_263-734	1,00E-20	92/338 (27%)	MBD2878651.1	1	1
**Imd pathway**							
PGRP-LB	NP_650079.1	Dg-540147_FR1_164-359	2,00E-36	76/162 (46%)		0	0
		Dg-540146_FR1_117-298	2,00E-36	76/162 (46%)			
		IrSigP-364299_FR6_987-1226	4,00E-24	58/170 (34%)			
		Dg-540150_FR4_273-459	1,00E-21	54/124 (43%)			
PGRP-SC	NP_610410.1	Dg-540147_FR1_164-359	7,00E-34	65/160 (40%)		0	0
		Dg-540146_FR1_117-298	7,00E-34	65/160 (40%)			
		IrSigP-364299_FR6_987-1226	2,00E-25	48/160 (30%)			
		Dg-540150_FR4_273-459	3,00E-23	49/128 (38%)			
PGRP-LC	NP_729468.2	Dg-540147_FR1_164-359	1,00E-33	68/162 (41%)		0	0
		Dg-540146_FR1_117-298	1,00E-33	68/162 (41%)			
		IrSigP-364299_FR6_987-1226	3,00E-26	54/156 (34%)			
		Dg-540150_FR4_273-459	3,00E-22	51/125 (40%)			
PGRP-LE	NP_573078.1	Dg-540147_FR1_164-359	2,00E-33	71/161 (44%)		0	0
		Dg-540146_FR1_117-298	2,00E-33	71/161 (44%)			
		Dg-540150_FR4_273-459	2,00E-21	52/125 (41%)			
		IrSigP-364299_FR6_987-1226	3,00E-20	41/117 (35%)			
imd	NP_573394.1	missing				0	0
tak1	NP_524080.1	Dg-625516_FR6_1422-2006	5,00E-67	150/374 (40%)	MBD2883593.1	2	1
		Dg-540567_FR1_999-1370	6,00E-35	88/231 (38%)	MBD2880322.1		

IKKgamma	NP_523856.2	missing				0	0
IKKbeta	NP_524751.3	Dg-442267_FR6_1351-2127	1,00E-35	119/375 (31%)	MBD2885575.1	3	2
		Dg-444853_FR2_344-1010	1,00E-35	119/375 (31%)	MBD2887170.1		
		Dg-823437_FR6_191-936	8,00E-27	91/283 (32%)	MBD2885364.1		
Fadd	NP_651006.1	missing				0	0
Dredd	NP_477251.3	Dg-598513_FR3_41-413	9,00E-17	86/308 (27%)	MBD2878667.1	7	4
		Dg-598516_FR3_41-422	9,00E-17	86/308 (27%)	MBD2885764.1		
		Dg-598535_FR2_222-590	4,00E-16	71/223 (31%)	MBD2885765.1		
		Dg-598533_FR3_41-446	4,00E-16	71/223 (31%)	MBD2885765.1		
		Dg-971248_FR1_366-698	8,00E-10	71/261 (27%)	MBD2875414.1		
		Dg-559693_FR4_576-1463	2,00E-05	33/137 (24%)	MBD2880927.1		
		Dg-559695_FR1_592-1466	2,00E-05	33/137 (24%)	MBD2880926.1		
Relish	NP_477094.1	missing				0	0
Caspar	NP_611080.1	missing				0	0
**RNAi pathway**							
Dicer-1	NP_524453	IrSigP-590221_FR1_364-2293	e-157	293/533 (54%)	MBD2887739.1	2	1
		Dg-590223_FR1_2376-2596	3,00E-64	128/197 (64%)	MBD2890853.1		
Dicer-2	NP_523778	Dg-575620_FR6_62-1581	6,00E-55	256/1059 (24%)	MBD2876567.1	1	1
Drosha	NP_477436	IrSigP-360170_FR2_1-1800	0	607/1247 (48%)	"MBD2878995.1	1	1
Pasha	NP_651879					0	0
R2D2	NP_609152					0	0
XPO5						0	0
Argonaute-1	NP_725341	Dg-708394_FR3_85-1031	0	715/904 (79%)	MBD2877359.1	6	5
		Dg-708392_FR1_92-1020	0	707/906 (78%)	MBD2877356.1		
		Dg-632068_FR1_374-1365	e-143	343/942 (36%)	MBD2878837.1		
		Dg-632065_FR4_131-1166	e-142	342/945 (36%)	MBD2878839.1		
		Dg-571008_FR5_218-1092	e-140	314/854 (36%)	MBD2877547.1		
		Dg-400391_FR1_369-1469	e-108	308/974 (31%)	MBD2875929.1		
Argonaute-2	NP_648775					0	0
Loquacious	NP_723813	Dg-1131049_FR2_355-836	9,00E-55	146/395 (36%)	MBD2882785.1	2	2
		Dg-364025_FR4_2135-2633	3,00E-05	36/136 (26%)	MBD2881328.1		
