# Supplementary material for: Blood-feeding adaptations and virome assessment of the poultry red mite Dermanyssus gallinae guided by RNA-seq
Source: Commun Biol. 2023 May 13;6:517. doi: 10.1038/s42003-023-04907-x (PMC10183022; doi:10.1038/s42003-023-04907-x)
Supplement: Supplementary file 2 — Description of Additional Supplementary Files [file 42003_2023_4907_MOESM2_ESM.pdf]

Description of Additional Supplementary Files

File name: Supplementary Data S1

Description: The source data behind the graphs in the paper.

File name: Supplementary Data S2

Description: Accession numbers of cysLGIC protein sequences
